# Supplementary material for: Inferring mitochondrial and cytosolic metabolism by coupling isotope tracing and deconvolution
Source: Nat Commun. 2023 Nov 18;14:7525. doi: 10.1038/s41467-023-42824-z (PMC10657349; doi:10.1038/s41467-023-42824-z)
Supplement: Supplementary file 1 — Supplementary Information [file 41467_2023_42824_MOESM1_ESM.pdf]

Supplementary Information

Inferring mitochondrial and cytosolic metabolism by coupling isotope tracing and deconvolution

Stern et al.

Supplementary Figures

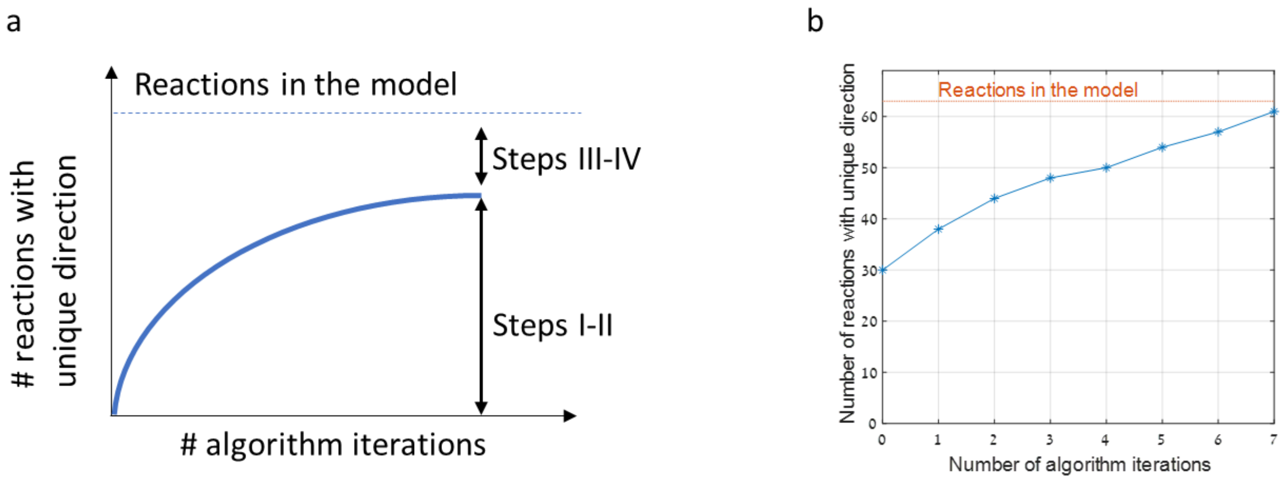

Figure S1: Determining flux directionalities via CODE-MFA. (a) Illustration of the number of reactions whose flux is uniquely determined throughout the different steps of CODE-MFA. (b) Number of reactions whose direction of net flux is uniquely inferred across CODE-MFA iterations in HeLa cells.

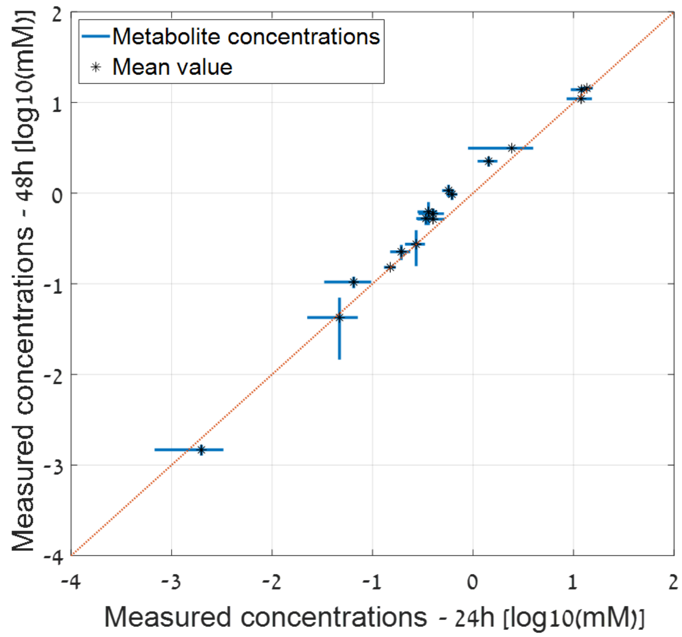

Figure S2: intracellular metabolite concentrations made after 24-hour vs. 48-hour incubation in HeLa cells (data are presented as mean values $\pm SD$, n=3 independent biological replicates).

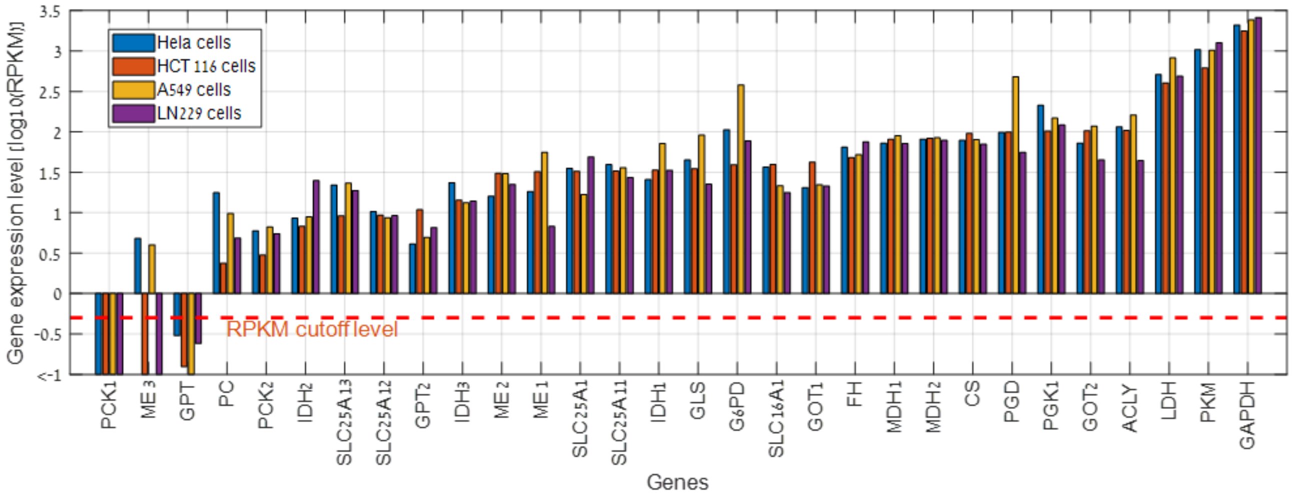

Figure S3: Gene expression levels obtained from the Broad Institute Cancer Cell Line Encyclopedia (CCLE), with 0.5 RPKM value as the cutoff to determine non-expressed genes.

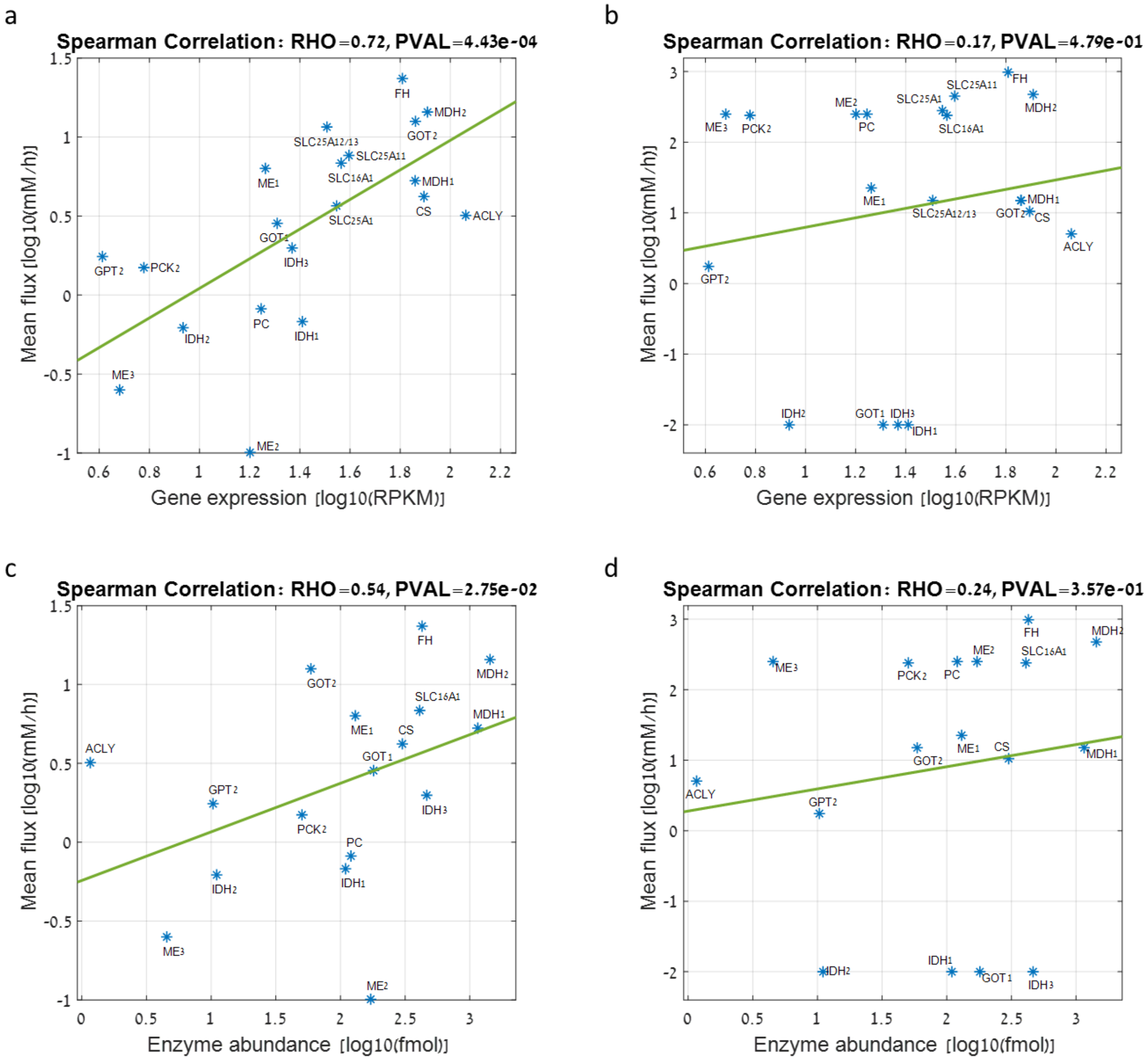

Figure S4: Correlation between gene expression and enzyme abundance levels with most probable net flux inferred by CODE-MFA in HeLa cells. (a) Gene expression levels and most probable net flux inferred by CODE-MFA through the corresponding enzyme. (b) Gene expression levels and most probable net flux inferred by MFA through the corresponding enzyme. (c) Measured enzyme abundance[47] and most probable net fluxes inferred by CODE-MFA (d) Measured enzyme abundance[88] and most probable net fluxes inferred by MFA

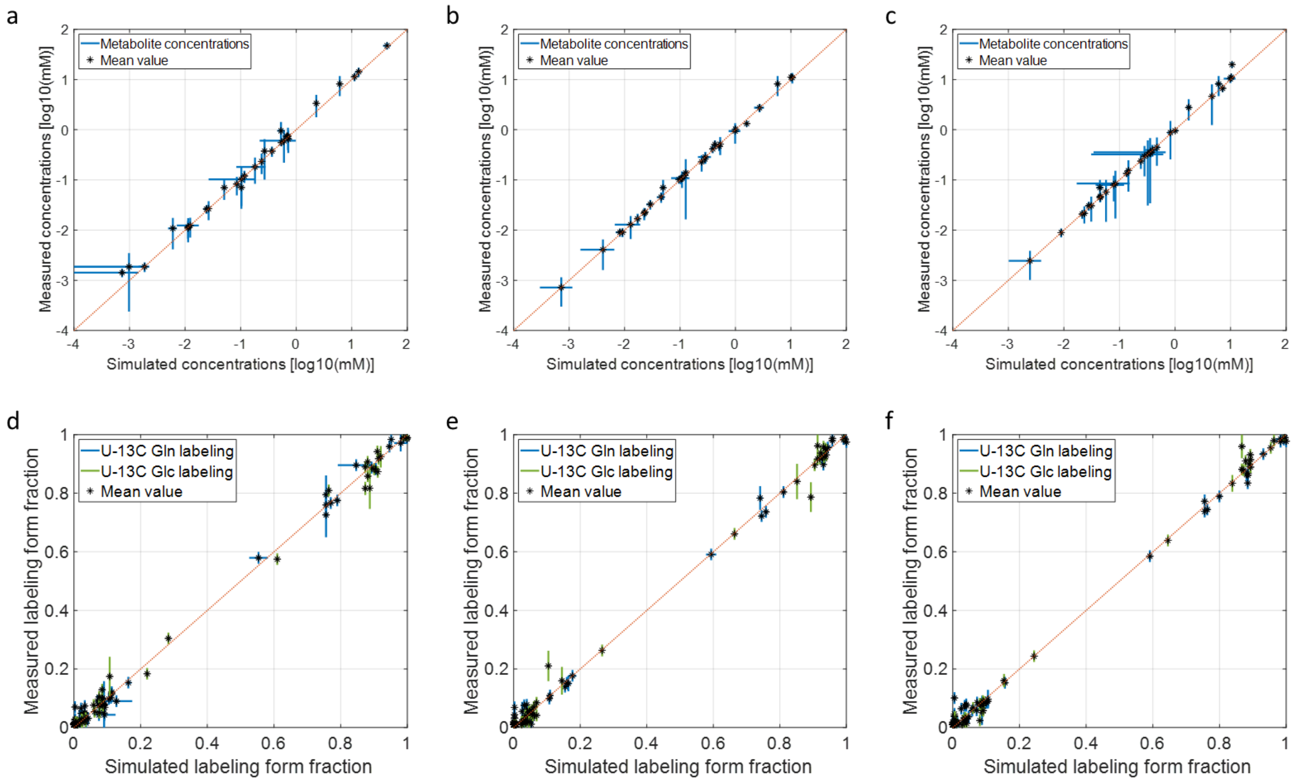

Figure S5: The fit between compartmentalized CODE-MFA predictions and total cellular measurements. (a-c) The fit been simulated total cellular metabolite concentration (i.e. convolution of simulated mitochondrial and cytosolic labeling; x-axis) and measurements (y-axis; data are presented as mean values ±SD, n=3 independent biological replicates) in HCT116 (a), A549 (b), and LN229 (c). (d-f) The fit been simulated total cellular metabolite isotopic labeling (x-axis) and experimental measurements (y-axis; data are presented as mean values ±SD, n=3 independent biological replicates) in HCT116 (d), A549 (e), and LN229 (f).

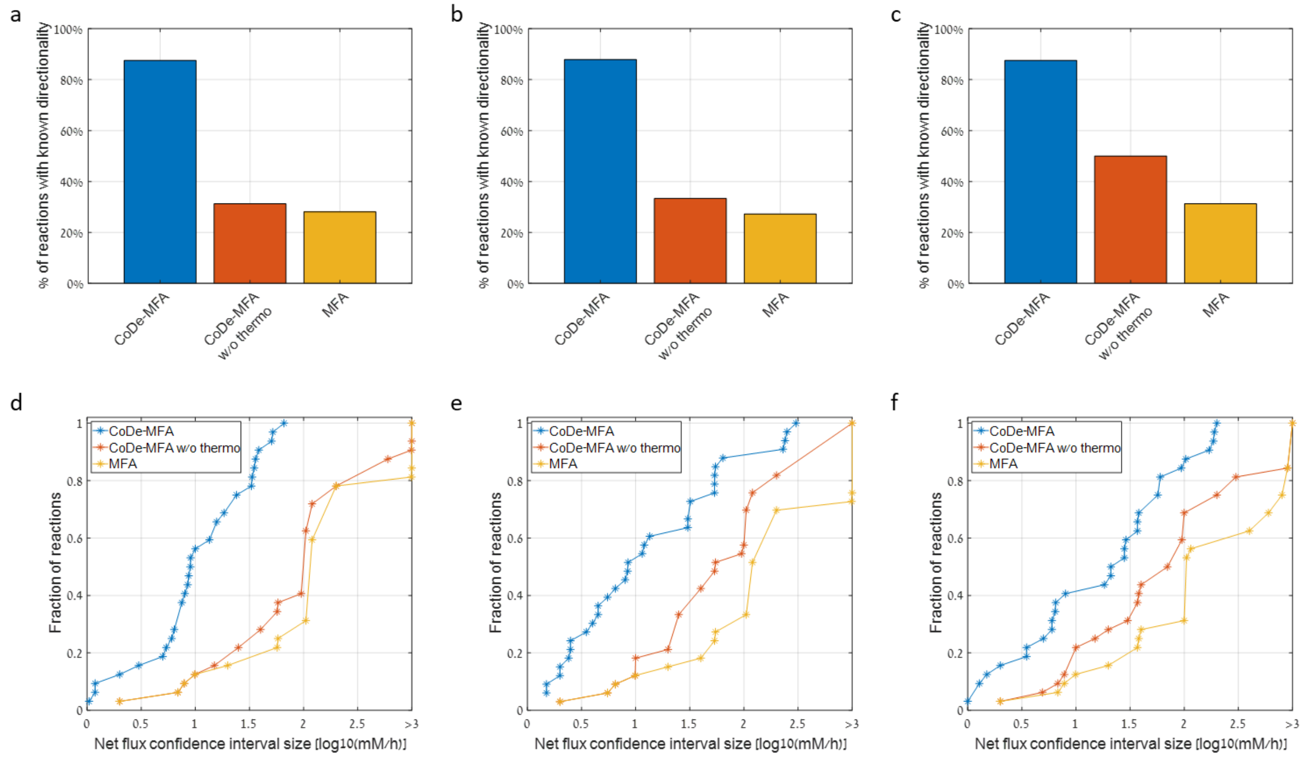

Figure S6: CODE-MFA performance in inferring compartmentalized fluxes in HCT116, A549, and LN229. (a,b,c) Percentage of reactions in the model whose direction of net flux is inferred by CODE-MFA versus with MFA and MFA without thermodynamics considerations in HCT116 (a), A549 (b), and LN229 (c). (d,e,f) Cumulative distribution of reaction net flux confidence interval sizes inferred by CODE-MFA versus MFA and CoDe-MFA without thermodynamic considerations in HCT116 (d), A549 (e), and LN229 (f).

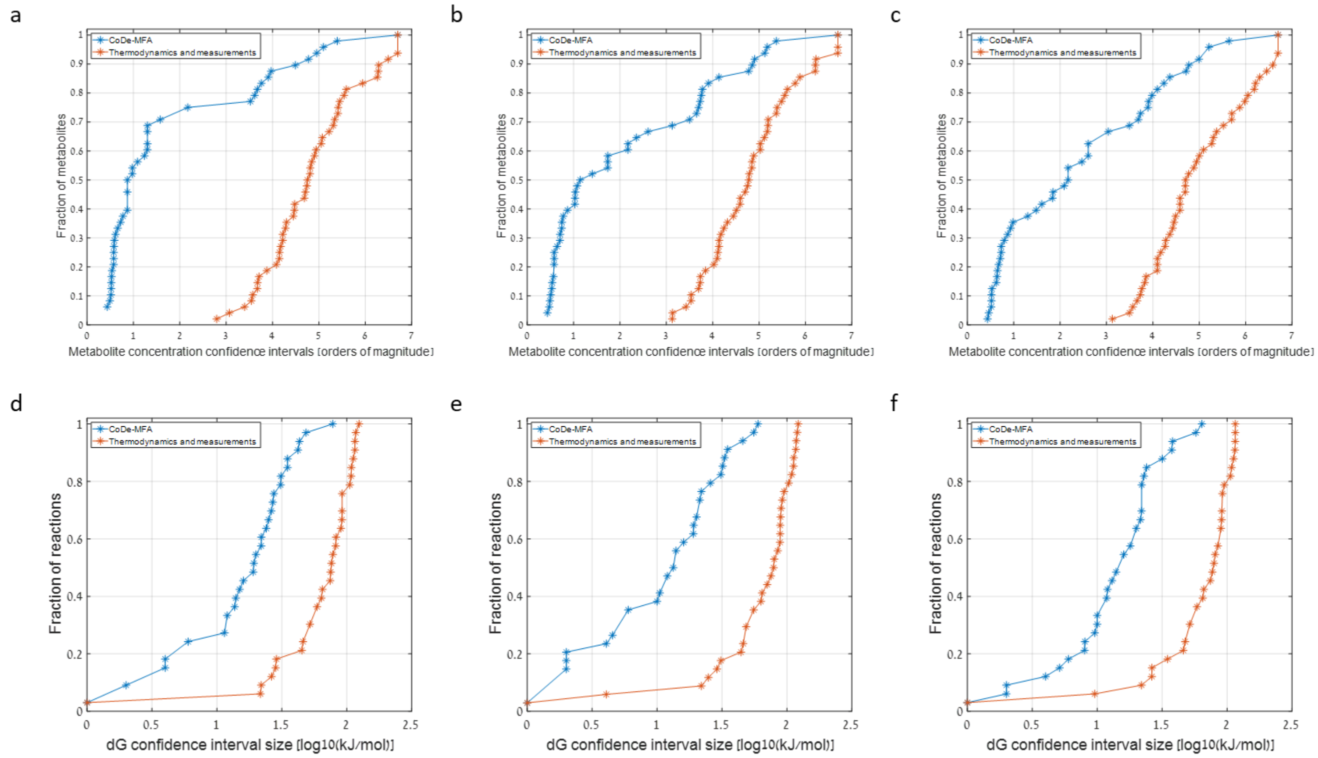

Figure S7: CODE-MFA performance in inferring compartmentalized metabolite concentrations and Gibbs free energy in HCT116, A549, and LN229. (a,b,c) Cumulative distribution of reaction metabolite concentration confidence interval sizes inferred by CODE-MFA versus with strictly thermodynamic analysis in HCT116 (a), A549 (b), and LN229 (c). (d,e,f) Cumulative distribution of reaction Gibbs energy confidence interval sizes inferred by CODE-MFA versus with strictly thermodynamic analysis in HCT116 (d), A549 (e), and LN229 (f).

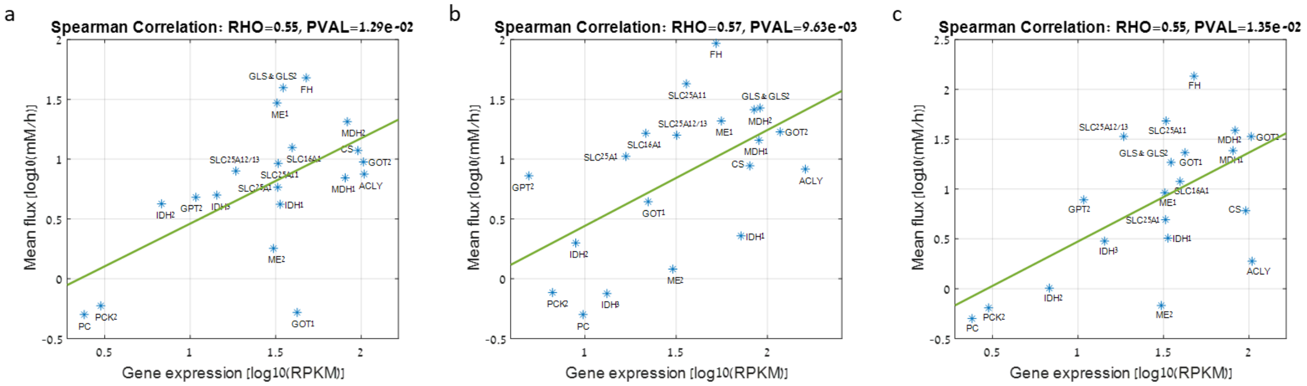

Figure S8: Correlation between CODE-MFA most probable net flux estimates and gene expression levels in HCT116 (a), A549 (b), and LN229 (c).

Supplementary Table 1. A compartmentalized network model of central metabolism used for CoDe-MFA - Hela cells

Reaction #	Reaction desctiption	Reaction type	Carbon mapping	Flux lower bound [mM/h]	Flux upper bound [mM/h]	Standard Gibbs free energy (kJ/mol)
v1	Glutamine_Media => Glutamine_CY	Uptake/Secretion	abcde => abcde	81	82	
v2	Glutamate_CY => Glutamate_Media	Uptake/Secretion	abcde => abcde	0	70	
v3	Glucose_Media => Glc6P_CY	Uptake/Secretion	abcdef => abcdef	111	355	
v4	Lactate_CY => Lactate_Media	Uptake/Secretion	abc => abc	506	643	
v5	Pyruvate_CY => Pyruvate_Media	Uptake/Secretion	abc => abc	3.5	4.8	
v13	Aspartate_CY => Fumarate_CY	Others	abcd => abcd	2	4	
v16	R5P_CY => R5P_CY_Pool	Demand	abcde => abcde	2	4	
v17	Acetyl_CoA_CY => Fatty_Acid	Demand	ab => ab	0	10	
v18	CarAsp_CY => Pyrimidines	Demand	abcde => abcde	2	4	
v19	Asparagine_CY => Asparagine_CY_Pool	Demand	abcd => abcd	1	3	
v21	Proline_MT => Proline_MT_Pool	Demand	abcde => abcde	1	3	
v51	Glutamine_CY <=> Glutamine_MT	Others	abcde => abcde	-Inf	Inf	
v52.1	Glutamate_CY <=> Glutamate_MT	Others	abcde => abcde	-Inf	Inf	
v53.1	AKG_CY + Malate_MT => AKG_MT + Malate_CY	Others	abcde + fghi => abcde + fghi	-Inf	Inf	0
v53.2	AKG_MT + Glutathione_CY => AKG_CY + Glutathione_MT	Others	abcde => abcde	-Inf	Inf	-14.4
v55	Fumarate_MT <=> Fumarate_CY	Others	abcd => abcd	-Inf	Inf	
v56.1	Malate_MT + Citrate_CY => Malate_CY + Citrate_MT	Others	abcd + efghij => abcd + efghij	-Inf	Inf	14.5
v57	Aspartate_MT + Glutamate_CY => Aspartate_CY + Glutamate_MT	Others	abcd + efghi => abcd + efghi	-Inf	Inf	-17.43
v58	Alanine_MT => Alanine_CY	Others	abc => abc	-Inf	Inf	0.07
v59	Pyruvate_MT => Pyruvate_CY	Others	abc => abc	-Inf	Inf	2.9
v60	PEP_CY + Citrate_MT => PEP_MT + Citrate_CY	Others	abc + defghi => abc + defghi	-Inf	Inf	-0.04
v101	Glutamine_CY => Glutamine_CY_Pool	Demand	abcde => abcde	1	3	
v102	Glutamine_CY <=> Glutamate_CY	Others	abcde => abcde	-Inf	Inf	
v105	Glutamate_CY => Glutamate_CY_Pool	Demand	abcde => abcde	1	3	
v106.1	Glutamate_CY <=> AKG_CY	Others	abcde => abcde	-Inf	Inf	
v107	Citrate_CY + NADP_CY => AKG_CY + NADPH_CY + CO2	Others	abcdef => abcde + f	-Inf	Inf	-3.1
v108	Citrate_CY + ATP_CY + CoA_CY => Acetyl_CoA_CY + OAA_CY + ADP_CY + Orthophosphate_CY	Others	abcdef => ed + fcba	-Inf	Inf	-8.9
v109	Fumarate_CY => Malate_CY	Others	abcd => abcd or dcba	-Inf	Inf	-3.5
v111	Malate_CY + NAD_CY => OAA_CY + NADH_CY	Others	abcd => abcd	-Inf	Inf	30.3
v112	Malate_CY + NADP_CY => Pyruvate_CY + CO2 + NADPH_CY	Others	abcd => abc + d	-Inf	Inf	-3.1
v113	OAA_CY + Glutamate_CY => Aspartate_CY + AKG_CY	Others	abcd + efghi => abcd + efghi	-Inf	Inf	-2.9
v114	Aspartate_CY + CO2 => CarAsp_CY	Others	abcd + e => abcde	0	Inf	
v115	Aspartate_CY => Asparagine_CY	Others	abcd => abcd	0	Inf	
v116	Aspartate_CY => Aspartate_CY_Pool	Demand	abcd => abcd	1	3	
v117	Alanine_CY => Alanine_CY_Pool	Demand	abc => abc	1	3	
v118	Pyruvate_CY + NADH_CY => Lactate_CY + NAD_CY	Others	abc => abc	0	Inf	-27.5
v119	PEP_CY + ADP_CY => Pyruvate_CY + ATP_CY	Others	abc => abc	0	Inf	-27.7
v120	3PG_CY => PEP_CY	Others	abc => abc	0	Inf	
v121	13BPG_CY + ADP_CY => 3PG_CY + ATP_CY	Others	abc => abc	0	Inf	-18.5
v122	G3P_CY + NAD_CY + Orthophosphate_CY => 13BPG_CY + NADH_CY	Others	abc => abc	0	Inf	24.9
v123	Glc6P_CY => G3P_CY + G3P_CY	Others	abcdef => abc + fed	0	Inf	
v124	Glc6P_CY + NADP_CY => 6phosphogluconate_CY + NADPH_CY	Others	abcdef => abcdef	0	Inf	-23.2
v125	6phosphogluconate_CY + NADP_CY => R5P_CY + NADPH_CY + CO2	Others	abcdef => abcde + f	-Inf	Inf	-7.8
v201	Glutamine_MT => Glutamine_MT_Pool	Demand	abcde => abcde	1	3	
v202	Glutamine_MT => Glutamate_MT + NH3	Others	abcde => abcde	-Inf	Inf	-28.6
v203	Glutamate_MT => Proline_MT	Others	abcde => abcde	0	Inf	
v204	Glutamate_MT => Glutamate_MT_Pool	Demand	abcde => abcde	1	3	
v205.1	Glutamate_MT <=> AKG_MT	Others	abcde => abcde	-Inf	Inf	
v206	AKG_MT + CO2 + NADPH_MT => Citrate_MT + NADP_MT	Others	abcde + f => abcdef	-Inf	Inf	3.1
v207	AKG_MT + CO2 + NADH_MT => Citrate_MT + NAD_MT	Others	abcde + f => abcdef	-Inf	Inf	4.1
v208	Acetyl_CoA_MT + OAA_MT => Citrate_MT + CoA_MT	Others	ab + cdef => fedbac	-Inf	Inf	-37.5
v209	AKG_MT => CO2 + Fumarate_MT	Others	abcde => abcd + e	0	Inf	
v210	Fumarate_MT => Malate_MT	Others	abcd => abcd or dcba	-Inf	Inf	-3.4
v212	Malate_MT + NAD_MT => OAA_MT + NADH_MT	Others	abcd => abcd	-Inf	Inf	27.5
v213.1	Malate_MT + NAD_MT => Pyruvate_MT + CO2 + NADH_MT	Others	abcd => abc + d	-Inf	Inf	-4.1
v213.2	Malate_MT + NADP_MT => Pyruvate_MT + CO2 + NADPH_MT	Others	abcd => abc + d	-Inf	Inf	-3.1
v214	Aspartate_MT + AKG_MT => OAA_MT + Glutamate_MT	Others	abcd + efghi => abcd + efghi	-Inf	Inf	2.9
v215	Aspartate_MT => Aspartate_MT_Pool	Demand	abcd => abcd	1	3	
v216	Alanine_MT => Alanine_MT_Pool	Demand	abc => abc	1	3	
v217	Pyruvate_MT + Glutamate_MT => Alanine_MT + AKG_MT	Others	abc + defgh => abc + defgh	-Inf	Inf	-0.4
v218	PEP_MT + CO2 + GDP_MT => OAA_MT + GTP_MT	Others	abc + d => abcd	-Inf	Inf	4
v219	Pyruvate_MT + NAD_MT + CoA_MT => CO2 + Acetyl_CoA_MT + NADH_MT	Others	abc => a + bc	0	Inf	-35.3
v220	Pyruvate_MT + CO2 + ATP_MT => OAA_MT + Orthophosphate_MT + ADP_MT	Others	abc + d => abcd	0	Inf	-6.4

Supplementary Table 2. The fractional labeling of metabolites when feeding HeLa cells with [U-13C]-glucose and [U-13C]-glutamine for 24 h.

Metabolite	Mass-isotopomer	Glutamine (measured mean - after natural abundance and impurity correction)	Glutamine (measured SD)	Glutamine (simulated best match)	Glucose (measured mean - after natural abundance and impurity correction)	Glucose (measured SD)	Glucose (simulated best match)	Lactate (measured mean - after natural abundance and impurity correction)	Lactate (measured SD)
6phosphogluconate	M+0	1	0	1	0.07	0.038	0		
6phosphogluconate	M+1	0	0	0	0	0.002	0		
6phosphogluconate	M+2	0	0	0	0	0	0		
6phosphogluconate	M+3	0	0	0	0	0	0		
6phosphogluconate	M+4	0	0	0	0	0.001	0		
6phosphogluconate	M+5	0	0	0	0.05	0.003	0.05		
6phosphogluconate	M+6	0	0	0	0.89	0.04	0.95		
R5P	M+0	1	0.008	1	0	0.004	0		
R5P	M+1	0	0.008	0	0	0	0		
R5P	M+2	0	0	0	0.01	0.002	0		
R5P	M+3	0	0	0	0.01	0.002	0		
R5P	M+4	0	0	0	0	0.002	0		
R5P	M+5	0	0	0	0.98	0.01	1		
Lactate	M+0	0.99	0.001	0.99	0.04	0.002	0.01	0.965	0.003
Lactate	M+1	0	0.001	0	0	0	0	0.01	0
Lactate	M+2	0	0	0	0	0.002	0	0	0
Lactate	M+3	0.01	0	0.01	0.96	0.001	0.99	0.025	0.004
PEP	M+0	1	0.003	0.99	0.02	0.009	0.01		
PEP	M+1	0	0.003	0	0	0	0		
PEP	M+2	0	0	0	0	0	0		
PEP	M+3	0	0	0.01	0.98	0.009	0.99		
Pyruvate	M+0	0.99	0.004	0.99	0.02	0.003	0.01	0.975	0.001
Pyruvate	M+1	0	0.002	0	0	0	0	0.015	0.001
Pyruvate	M+2	0	0	0	0	0.002	0	0	0
Pyruvate	M+3	0.01	0.005	0.01	0.98	0.002	0.99	0.01	0.001
Alanine	M+0	0.99	0.001	0.99	0.13	0.008	0.13		
Alanine	M+1	0	0.001	0	0	0	0		
Alanine	M+2	0	0	0	0	0.003	0		
Alanine	M+3	0.01	0.001	0.01	0.87	0.009	0.87		
Citrate	M+0	0.12	0.043	0.12	0.2	0.043	0.2		
Citrate	M+1	0	0.003	0	0.01	0.002	0		
Citrate	M+2	0.03	0.003	0.02	0.64	0.032	0.67		
Citrate	M+3	0.12	0.006	0.12	0.03	0.004	0.01		
Citrate	M+4	0.58	0.029	0.58	0.02	0.001	0.02		
Citrate	M+5	0.15	0.008	0.15	0.1	0.007	0.1		
Citrate	M+6	0.01	0.002	0.01	0	0	0		
AKG	M+0	0.03	0	0	0.99	0.003	0.97		
AKG	M+1	0	0	0	0	0.001	0		
AKG	M+2	0	0.013	0	0.01	0.003	0.02		
AKG	M+3	0.01	0.003	0.02	0	0	0		
AKG	M+4	0	0.004	0	0	0	0		
AKG	M+5	0.95	0.012	0.97	0	0.001	0		
Glutamine	M+0	0.01	0.001	0	1	0	1		
Glutamine	M+1	0	0	0	0	0	0		
Glutamine	M+2	0	0	0	0	0	0		
Glutamine	M+3	0	0	0	0	0	0		
Glutamine	M+4	0	0	0	0	0	0		
Glutamine	M+5	0.99	0.001	1	0	0	0		
Glutamate media	M+0	0.03	0.002	0	0.98	0	0.97		

Glutamate media	M+1	0	0	0	0	0	0
Glutamate media	M+2	0	0	0	0.02	0.001	0.02
Glutamate media	M+3	0.02	0	0.02	0	0	0
Glutamate media	M+4	0	0	0	0	0	0
Glutamate media	M+5	0.95	0.002	0.97	0	0	0
Glutamate	M+0	0.03	0.002	0	0.98	0.001	0.98
Glutamate	M+1	0	0	0	0	0.001	0
Glutamate	M+2	0	0	0	0.01	0	0.02
Glutamate	M+3	0.01	0	0.01	0	0	0
Glutamate	M+4	0	0.001	0	0	0	0
Glutamate	M+5	0.96	0.002	0.98	0	0	0
Fumarate	M+0	0.16	0.032	0.12	0.87	0.02	0.87
Fumarate	M+1	0.01	0.01	0	0.01	0.009	0
Fumarate	M+2	0.01	0.001	0.02	0	0.005	0.02
Fumarate	M+3	0.14	0.008	0.15	0.11	0.012	0.11
Fumarate	M+4	0.68	0.016	0.71	0	0.001	0
Asparagine	M+0	0.15	0.006	0.13	0.86	0.01	0.85
Asparagine	M+1	0.02	0.008	0	0.01	0.002	0
Asparagine	M+2	0	0	0.02	0.02	0.001	0.02
Asparagine	M+3	0.14	0.003	0.18	0.12	0.01	0.12
Asparagine	M+4	0.69	0.004	0.66	0	0	0
Aspartate	M+0	0.14	0	0.13	0.86	0.003	0.85
Aspartate	M+1	0	0.001	0	0	0.001	0
Aspartate	M+2	0.03	0.001	0.02	0.02	0	0.02
Aspartate	M+3	0.21	0.002	0.18	0.11	0.002	0.12
Aspartate	M+4	0.62	0.003	0.67	0	0	0
CarAsp	M+0	0.13	0.01	0.13	0.88	0.017	0.85
CarAsp	M+1	0	0.003	0	0	0.008	0
CarAsp	M+2	0.01	0.004	0.02	0	0	0.02
CarAsp	M+3	0.17	0.012	0.18	0.11	0.009	0.12
CarAsp	M+4	0.69	0.02	0.66	0	0	0
CarAsp	M+5	0	0.001	0	0	0	0
Malate	M+0	0.12	0.003	0.11	0.89	0.009	0.88
Malate	M+1	0	0.001	0	0	0.001	0
Malate	M+2	0.02	0.002	0.02	0.02	0.001	0.02
Malate	M+3	0.18	0.001	0.19	0.09	0.009	0.1
Malate	M+4	0.67	0.004	0.68	0	0	0
Palmitic acid	M+0	0.63	0.018		0.59	0.01	
Palmitic acid	M+1	0.02	0.006		0.02	0.007	
Palmitic acid	M+2	0.08	0.004		0.03	0.002	
Palmitic acid	M+3	0	0.002		0	0.001	
Palmitic acid	M+4	0.08	0.009		0.03	0.002	
Palmitic acid	M+5	0	0.002		0	0	
Palmitic acid	M+6	0.07	0.008		0.04	0.003	
Palmitic acid	M+7	0	0		0	0	
Palmitic acid	M+8	0.05	0.001		0.05	0.004	
Palmitic acid	M+9	0	0.001		0	0.001	
Palmitic acid	M+10	0.04	0.003		0.08	0.001	
Palmitic acid	M+11	0	0.002		0	0.002	
Palmitic acid	M+12	0.02	0.001		0.08	0.004	
Palmitic acid	M+13	0	0		0	0.001	
Palmitic acid	M+14	0.01	0.001		0.06	0.002	
Palmitic acid	M+15	0	0		0	0.001	
Palmitic acid	M+16	0	0		0.02	0.002	
Acetyl-CoA	M+0	0.61	0.011		0.01	0.017	

Acetyl-CoA	M+1	0.05	0.002	0	0.003
Acetyl-CoA	M+2	0.32	0.003	0.03	0.001
Acetyl-CoA	M+3	0.02	0.006	0	0.004
Acetyl-CoA	M+4	0	0.01	0.01	0.004
Acetyl-CoA	M+5	0	0	0.36	0.005
Acetyl-CoA	M+6	0	0	0.06	0
Acetyl-CoA	M+7	0	0	0.43	0.021
Acetyl-CoA	M+8	0	0	0.06	0.01
Acetyl-CoA	M+9	0	0	0	0
Acetyl-CoA	M+10	0	0	0	0
Acetyl-CoA	M+11	0	0	0	0
Acetyl-CoA	M+12	0	0	0	0
Acetyl-CoA	M+13	0	0	0	0
Acetyl-CoA	M+14	0	0	0	0
Acetyl-CoA	M+15	0	0	0	0
Acetyl-CoA	M+16	0	0	0	0
Acetyl-CoA	M+17	0	0	0	0
Acetyl-CoA	M+18	0	0	0	0
Acetyl-CoA	M+19	0	0	0	0
Acetyl-CoA	M+20	0	0	0	0
Acetyl-CoA	M+21	0	0	0	0
Acetyl-CoA	M+22	0	0	0	0
Acetyl-CoA	M+23	0	0	0	0
CoA	M+0	0.86	0.028	0.07	0.006
CoA	M+1	0.06	0.011	0.01	0.006
CoA	M+2	0.07	0.006	0.01	0.002
CoA	M+3	0	0.005	0	0.004
CoA	M+4	0	0.001	0	0
CoA	M+5	0.01	0.018	0.74	0.003
CoA	M+6	0	0.007	0.11	0.018
CoA	M+7	0	0	0.06	0.005
CoA	M+8	0	0	0	0.011
CoA	M+9	0	0	0	0.011
CoA	M+10	0	0	0	0
CoA	M+11	0	0	0	0
CoA	M+12	0	0	0	0
CoA	M+13	0	0	0	0
CoA	M+14	0	0	0	0
CoA	M+15	0	0	0	0
CoA	M+16	0	0	0	0
CoA	M+17	0	0	0	0
CoA	M+18	0	0	0	0
CoA	M+19	0	0	0	0
CoA	M+20	0	0	0	0
CoA	M+21	0	0	0	0

Supplementary Table 3. The fractional labeling of metabolites when feeding HCT116 cells with [U-13C]-glucose and [U-13C]-glutamine for 24 h.

Metabolite	Mass-isotopomer	Glutamine (measured mean - after natural abundance and impurity correction)	Glutamine (measured SD)	Glutamine (simulated best match)	Glucose (measured mean - after natural abundance and impurity correction)	Glucose (measured SD)	Glucose (simulated best match)	Lactate (measured mean - after natural abundance and impurity correction)	Lactate (measured SD)
6phosphogluconate	M+0	1	0.004	1	0.05	0.007	0.06		
6phosphogluconate	M+1	0	0.004	0	0	0	0		
6phosphogluconate	M+2	0	0	0	0	0	0		
6phosphogluconate	M+3	0	0	0	0	0	0		
6phosphogluconate	M+4	0	0	0	0	0	0		
6phosphogluconate	M+5	0	0	0	0.03	0.004	0.02		
6phosphogluconate	M+6	0	0	0	0.92	0.003	0.91		
Lactate	M+0	0.99	0.003	0.95	0.12	0.007	0.1	0.98	0.002
Lactate	M+1	0	0.001	0	0	0	0	0.01	0
Lactate	M+2	0	0	0.01	0	0	0	0	0
Lactate	M+3	0.01	0.002	0.04	0.88	0.007	0.89	0.01	0.001
PEP	M+0	0.98	0.017	0.98	0.08	0.018	0.08		
PEP	M+1	0	0.002	0	0	0	0		
PEP	M+2	0	0	0	0	0	0		
PEP	M+3	0.02	0.019	0.02	0.92	0.018	0.92		
Pyruvate	M+0	0.96	0.006	0.95	0.17	0.034	0.1	0.97	0.006
Pyruvate	M+1	0.01	0.002	0	0	0.002	0	0.02	0.005
Pyruvate	M+2	0	0.003	0.01	0.01	0.001	0	0	0
Pyruvate	M+3	0.03	0.002	0.04	0.82	0.035	0.89	0.01	0.001
Alanine	M+0	0.9	0.001	0.9	0.18	0.007	0.18		
Alanine	M+1	0	0	0.01	0	0	0		
Alanine	M+2	0.01	0	0.01	0	0.001	0.01		
Alanine	M+3	0.09	0.001	0.08	0.81	0.006	0.81		
Citrate	M+0	0.02	0.017	0.02	0.25	0.007	0.25		
Citrate	M+1	0.02	0.001	0.01	0.01	0.002	0.01		
Citrate	M+2	0.09	0	0.08	0.62	0.004	0.64		
Citrate	M+3	0.09	0.001	0.1	0.02	0	0.01		
Citrate	M+4	0.58	0.011	0.58	0.08	0.004	0.07		
Citrate	M+5	0.15	0.008	0.15	0.02	0.001	0.03		
Citrate	M+6	0.04	0.002	0.06	0	0	0		
AKG	M+0	0.03	0.003	0	0.88	0.003	0.9		
AKG	M+1	0	0	0.01	0	0.003	0		
AKG	M+2	0.01	0	0.01	0.1	0.001	0.09		
AKG	M+3	0.07	0.001	0.09	0.01	0.001	0.01		
AKG	M+4	0	0	0	0.01	0	0.01		
AKG	M+5	0.89	0.004	0.89	0	0	0		
Glutamine	M+0	0.01	0.001	0	1	0.001	1		
Glutamine	M+1	0	0	0	0	0	0		
Glutamine	M+2	0	0	0	0	0	0		
Glutamine	M+3	0	0	0	0	0	0		
Glutamine	M+4	0	0	0	0	0	0		
Glutamine	M+5	0.99	0.001	1	0	0	0		
Glutamate media	M+0	0.07	0.002	0	0.94	0.01	0.91		
Glutamate media	M+1	0	0	0.01	0	0	0		
Glutamate media	M+2	0	0	0.01	0.05	0.008	0.08		
Glutamate media	M+3	0.04	0.001	0.08	0	0.004	0		
Glutamate media	M+4	0	0	0	0	0	0.01		
Glutamate media	M+5	0.88	0.003	0.9	0	0	0		
Glutamate	M+0	0.03	0.001	0	0.87	0.002	0.91		
Glutamate	M+1	0	0.001	0.01	0	0	0		
Glutamate	M+2	0.01	0	0.01	0.1	0.002	0.08		
Glutamate	M+3	0.08	0.002	0.08	0.01	0	0		
Glutamate	M+4	0	0.001	0	0.01	0	0.01		

Glutamate	M+5	0.88	0.003	0.9	0	0	0
Fumarate	M+0	0.07	0.003	0.02	0.91	0.01	0.88
Fumarate	M+1	0.01	0.003	0.01	0	0.001	0
Fumarate	M+2	0.07	0.006	0.1	0.08	0.007	0.09
Fumarate	M+3	0.08	0.005	0.09	0.02	0.006	0.03
Fumarate	M+4	0.78	0.009	0.78	0	0	0
Asparagine	M+0	0.07	0.006	0.03	0.89	0.005	0.87
Asparagine	M+1	0.01	0.001	0.01	0.01	0.001	0
Asparagine	M+2	0.08	0.041	0.09	0.08	0.004	0.09
Asparagine	M+3	0.12	0.004	0.11	0.02	0	0.03
Asparagine	M+4	0.73	0.038	0.75	0	0.001	0
Aspartate	M+0	0.05	0.001	0.03	0.82	0.011	0.87
Aspartate	M+1	0.01	0.001	0.01	0.02	0.004	0
Aspartate	M+2	0.08	0.001	0.09	0.13	0.007	0.09
Aspartate	M+3	0.1	0.004	0.11	0.03	0.001	0.03
Aspartate	M+4	0.76	0.004	0.75	0	0.001	0
CarAsp	M+0	0.05	0.007	0.03			
CarAsp	M+1	0	0	0.01			
CarAsp	M+2	0.03	0.033	0.09			
CarAsp	M+3	0.12	0.001	0.11			
CarAsp	M+4	0.8	0.033	0.75			
CarAsp	M+5	0	0	0			
Malate	M+0	0.05	0.003	0.02	0.86	0.005	0.87
Malate	M+1	0.01	0.001	0.01	0.01	0.002	0
Malate	M+2	0.08	0.006	0.09	0.1	0.002	0.09
Malate	M+3	0.1	0.001	0.1	0.02	0.001	0.03
Malate	M+4	0.77	0.007	0.77	0	0	0
Palmitic acid	M+0	0.94	0.003		0.95	0.002	
Palmitic acid	M+1	0	0.002		0	0	
Palmitic acid	M+2	0.02	0.001		0.01	0	
Palmitic acid	M+3	0	0		0	0	
Palmitic acid	M+4	0.01	0.001		0	0	
Palmitic acid	M+5	0	0		0	0	
Palmitic acid	M+6	0.01	0.001		0.01	0	
Palmitic acid	M+7	0	0		0	0	
Palmitic acid	M+8	0.01	0.001		0.01	0	
Palmitic acid	M+9	0	0		0	0	
Palmitic acid	M+10	0.01	0		0.01	0.001	
Palmitic acid	M+11	0	0		0	0	
Palmitic acid	M+12	0	0		0.01	0	
Palmitic acid	M+13	0	0		0	0	
Palmitic acid	M+14	0	0		0	0	
Palmitic acid	M+15	0	0		0	0	
Palmitic acid	M+16	0	0		0	0	
Acetyl-CoA	M+0	0.63	0.017		0.05	0.004	
Acetyl-CoA	M+1	0.05	0.008		0	0.002	
Acetyl-CoA	M+2	0.29	0.014		0.09	0.002	
Acetyl-CoA	M+3	0.02	0.002		0.01	0.004	
Acetyl-CoA	M+4	0.01	0.001		0.01	0.004	
Acetyl-CoA	M+5	0	0.001		0.26	0.007	
Acetyl-CoA	M+6	0	0.001		0.05	0.007	
Acetyl-CoA	M+7	0	0		0.43	0.015	
Acetyl-CoA	M+8	0	0		0.05	0.002	
Acetyl-CoA	M+9	0	0		0.03	0.001	
Acetyl-CoA	M+10	0	0.001		0	0.001	
Acetyl-CoA	M+11	0	0		0	0.001	
Acetyl-CoA	M+12	0	0		0	0	
Acetyl-CoA	M+13	0	0		0	0	
Acetyl-CoA	M+14	0	0		0	0	
Acetyl-CoA	M+15	0	0		0	0	

Acetyl-CoA	M+16	0	0	0	0
Acetyl-CoA	M+17	0	0	0	0
Acetyl-CoA	M+18	0	0	0	0
Acetyl-CoA	M+19	0	0	0	0
Acetyl-CoA	M+20	0	0	0	0
Acetyl-CoA	M+21	0	0	0	0
Acetyl-CoA	M+22	0	0	0	0
Acetyl-CoA	M+23	0	0	0	0
CoA	M+0	0.86	0.004	0.13	0.011
CoA	M+1	0.07	0.001	0.01	0.003
CoA	M+2	0.06	0.001	0.02	0.005
CoA	M+3	0	0.005	0.02	0.004
CoA	M+4	0	0.001	0.01	0.003
CoA	M+5	0.01	0.001	0.67	0.017
CoA	M+6	0	0.001	0.08	0.003
CoA	M+7	0	0	0.04	0.005
CoA	M+8	0	0	0.01	0.001
CoA	M+9	0	0	0	0
CoA	M+10	0	0	0	0
CoA	M+11	0	0	0	0
CoA	M+12	0	0	0	0
CoA	M+13	0	0.002	0	0
CoA	M+14	0	0.002	0	0
CoA	M+15	0	0	0	0
CoA	M+16	0	0	0	0
CoA	M+17	0	0	0	0
CoA	M+18	0	0	0	0
CoA	M+19	0	0	0	0
CoA	M+20	0	0	0	0
CoA	M+21	0	0	0	0

Supplementary Table 4. The fractional labeling of metabolites when feeding A549 cells with [U-13C]-glucose and [U-13C]-glutamine for 24 h.

Metabolite	Mass-isotopomer	Glutamine (measured mean - after natural abundance and impurity correction)	Glutamine (measured SD)	Glutamine (simulated best match)	Glucose (measured mean - after natural abundance and impurity correction)	Glucose (measured SD)	Glucose (simulated best match)	Lactate (measured mean - after natural abundance and impurity correction)	Lactate (measured SD)
6phosphogluconate	M+0	0.97	0.007	1	0.08	0.007	0.07		
6phosphogluconate	M+1	0	0.004	0	0	0.002	0		
6phosphogluconate	M+2	0	0	0	0	0	0		
6phosphogluconate	M+3	0	0	0	0	0	0		
6phosphogluconate	M+4	0	0	0	0	0	0		
6phosphogluconate	M+5	0	0.002	0	0.02	0.004	0.03		
6phosphogluconate	M+6	0.02	0.004	0	0.9	0.007	0.9		
Lactate	M+0	0.99	0	0.96	0.1	0.021	0.1	0.98	0.001
Lactate	M+1	0	0	0	0	0.001	0	0.01	0
Lactate	M+2	0	0	0.01	0	0.002	0	0	0
Lactate	M+3	0.01	0.001	0.03	0.9	0.021	0.89	0.01	0.001
PEP	M+0	0.99	0.006	1	0.04	0.006	0.07		
PEP	M+1	0	0.004	0	0	0.001	0		
PEP	M+2	0	0	0	0	0	0		
PEP	M+3	0.01	0.005	0	0.96	0.007	0.93		
Pyruvate	M+0	0.98	0.003	0.96	0.21	0.026	0.1	0.97	0.001
Pyruvate	M+1	0.01	0.002	0	0	0	0	0.03	0.001
Pyruvate	M+2	0	0	0.01	0	0.001	0	0	0
Pyruvate	M+3	0.01	0.002	0.03	0.79	0.025	0.89	0.003	0.002
Alanine	M+0	0.96	0.006	0.95	0.16	0.024	0.16		
Alanine	M+1	0	0.004	0	0	0.004	0		
Alanine	M+2	0	0.001	0.01	0	0.006	0		
Alanine	M+3	0.04	0.003	0.04	0.84	0.03	0.84		
Citrate	M+0	0.03	0.045	0.03	0.26	0.036	0.26		
Citrate	M+1	0.01	0.003	0	0.01	0.001	0		
Citrate	M+2	0.05	0.002	0.04	0.66	0.033	0.67		
Citrate	M+3	0.11	0.007	0.11	0.01	0.001	0.01		
Citrate	M+4	0.59	0.024	0.6	0.04	0.002	0.03		
Citrate	M+5	0.18	0.007	0.18	0.02	0.001	0.03		
Citrate	M+6	0.03	0.003	0.04	0	0	0		
AKG	M+0	0.03	0.002	0	0.92	0.002	0.93		
AKG	M+1	0	0.001	0	0	0.003	0		
AKG	M+2	0	0	0.01	0.07	0.003	0.06		
AKG	M+3	0.04	0.001	0.06	0	0.001	0		
AKG	M+4	0	0.001	0	0	0	0		
AKG	M+5	0.92	0.001	0.93	0	0	0		
Glutamine	M+0	0.01	0.001	0	1	0.001	0.99		
Glutamine	M+1	0	0	0	0	0.001	0		
Glutamine	M+2	0	0	0	0	0	0.01		
Glutamine	M+3	0	0	0.01	0	0	0		
Glutamine	M+4	0	0.001	0	0	0	0		
Glutamine	M+5	0.99	0	0.99	0	0	0		
Glutamate media	M+0	0.07	0.001	0	0.95	0.001	0.94		
Glutamate media	M+1	0	0	0	0	0.001	0		
Glutamate media	M+2	0	0	0.01	0.04	0.001	0.06		
Glutamate media	M+3	0.03	0.001	0.06	0	0	0		
Glutamate media	M+4	0	0.001	0	0	0	0		
Glutamate media	M+5	0.9	0	0.93	0	0	0		

Glutamate	M+0	0.03	0.001	0	0.93	0.001	0.94
Glutamate	M+1	0	0	0	0	0	0
Glutamate	M+2	0	0	0.01	0.06	0.001	0.05
Glutamate	M+3	0.04	0	0.05	0	0	0
Glutamate	M+4	0	0.001	0	0	0	0
Glutamate	M+5	0.92	0.001	0.93	0	0	0
Fumarate	M+0	0.06	0.016	0.03	0.94	0.003	0.92
Fumarate	M+1	0.01	0.001	0	0.01	0.003	0
Fumarate	M+2	0.04	0.002	0.05	0.05	0.003	0.05
Fumarate	M+3	0.1	0.01	0.11	0	0	0.02
Fumarate	M+4	0.8	0.004	0.81	0	0	0
Aspartate	M+0	0.08	0.001	0.04	0.92	0.002	0.91
Aspartate	M+1	0	0.001	0	0	0.001	0
Aspartate	M+2	0.04	0.001	0.05	0.05	0.001	0.05
Aspartate	M+3	0.15	0.002	0.16	0.02	0.002	0.03
Aspartate	M+4	0.72	0.001	0.74	0	0	0
CarAsp	M+0	0.06	0.009	0.04	0.98	0.027	0.92
CarAsp	M+1	0	0	0	0	0.009	0
CarAsp	M+2	0	0.002	0.05	0.01	0.014	0.05
CarAsp	M+3	0.15	0.013	0.16	0.01	0.006	0.03
CarAsp	M+4	0.78	0.02	0.74	0	0	0
CarAsp	M+5	0	0	0	0	0	0
Malate	M+0	0.08	0.003	0.03	0.92	0.002	0.92
Malate	M+1	0	0	0	0	0.001	0
Malate	M+2	0.04	0.001	0.05	0.05	0.001	0.05
Malate	M+3	0.14	0.003	0.15	0.02	0.001	0.03
Malate	M+4	0.74	0.007	0.76	0	0	0
Palmitic acid	M+0	0.74	0.003		0.75	0.015	
Palmitic acid	M+1	0	0.001		0	0.002	
Palmitic acid	M+2	0.08	0.002		0.01	0.001	
Palmitic acid	M+3	0	0		0	0	
Palmitic acid	M+4	0.07	0.001		0.01	0	
Palmitic acid	M+5	0	0		0	0	
Palmitic acid	M+6	0.05	0.001		0.02	0.001	
Palmitic acid	M+7	0	0		0	0	
Palmitic acid	M+8	0.03	0.001		0.04	0.001	
Palmitic acid	M+9	0	0		0	0.001	
Palmitic acid	M+10	0.02	0		0.06	0.003	
Palmitic acid	M+11	0	0		0	0.001	
Palmitic acid	M+12	0.01	0.001		0.06	0.004	
Palmitic acid	M+13	0	0		0	0.001	
Palmitic acid	M+14	0	0		0.04	0.003	
Palmitic acid	M+15	0	0		0	0.001	
Palmitic acid	M+16	0	0		0.01	0.001	
Acetyl-CoA	M+0	0.57	0.019		0.03	0	
Acetyl-CoA	M+1	0.05	0.01		0	0.003	
Acetyl-CoA	M+2	0.33	0.017		0.06	0.002	
Acetyl-CoA	M+3	0.03	0.008		0.01	0.004	
Acetyl-CoA	M+4	0.02	0.001		0.02	0.004	
Acetyl-CoA	M+5	0	0.001		0.28	0.009	
Acetyl-CoA	M+6	0	0		0.02	0.005	
Acetyl-CoA	M+7	0	0		0.51	0.008	
Acetyl-CoA	M+8	0	0		0.03	0.001	
Acetyl-CoA	M+9	0	0		0.04	0.005	
Acetyl-CoA	M+10	0	0		0	0	

Acetyl-CoA	M+11	0	0	0	0
Acetyl-CoA	M+12	0	0	0	0
Acetyl-CoA	M+13	0	0	0	0
Acetyl-CoA	M+14	0	0	0	0
Acetyl-CoA	M+15	0	0	0	0
Acetyl-CoA	M+16	0	0	0	0
Acetyl-CoA	M+17	0	0	0	0
Acetyl-CoA	M+18	0	0	0	0
Acetyl-CoA	M+19	0	0	0	0
Acetyl-CoA	M+20	0	0	0	0
Acetyl-CoA	M+21	0	0	0	0
Acetyl-CoA	M+22	0	0	0	0
Acetyl-CoA	M+23	0	0	0	0
CoA	M+0	0.84	0.009	0.09	0.015
CoA	M+1	0.06	0.002	0.01	0.002
CoA	M+2	0.07	0.004	0.02	0.002
CoA	M+3	0	0.005	0.01	0.003
CoA	M+4	0	0.001	0	0.003
CoA	M+5	0.02	0.003	0.76	0.009
CoA	M+6	0	0	0.04	0.005
CoA	M+7	0	0.001	0.05	0.006
CoA	M+8	0	0	0	0.002
CoA	M+9	0	0	0	0
CoA	M+10	0	0	0	0
CoA	M+11	0	0	0	0
CoA	M+12	0	0	0	0
CoA	M+13	0	0	0	0
CoA	M+14	0	0	0	0
CoA	M+15	0	0	0	0
CoA	M+16	0	0	0	0
CoA	M+17	0	0	0	0
CoA	M+18	0	0	0	0
CoA	M+19	0	0	0	0
CoA	M+20	0	0	0	0
CoA	M+21	0	0	0	0

Supplementary Table 5. The fractional labeling of metabolites when feeding LN229 cells with [U-13C]-glucose and [U-13C]-glutamine for 24 h.

Metabolite	Mass-isotopomer	Glutamine (measured mean - after natural abundance and impurity correction)	Glutamine (measured SD)	Glutamine (simulated best match)	Glucose (measured mean - after natural abundance and impurity correction)	Glucose (measured SD)	Glucose (simulated best match)	Lactate (measured mean - after natural abundance and impurity correction)	Lactate (measured SD)
6phosphogluconate	M+0	0.98	0.003	1	0.03	0.004	0.04		
6phosphogluconate	M+1	0	0.001	0	0	0.001	0		
6phosphogluconate	M+2	0.02	0.002	0	0	0	0		
6phosphogluconate	M+3	0	0	0	0	0	0		
6phosphogluconate	M+4	0	0	0	0	0	0		
6phosphogluconate	M+5	0	0	0	0.01	0.002	0.01		
6phosphogluconate	M+6	0.01	0.002	0	0.96	0.006	0.95		
Lactate	M+0	0.99	0.001	0.98	0.11	0.012	0.05	0.98	0.002
Lactate	M+1	0	0.001	0	0	0	0	0.01	0.001
Lactate	M+2	0	0	0	0	0.001	0	0	0
Lactate	M+3	0.01	0	0.01	0.89	0.012	0.95	0.01	0.001
PEP	M+0	1	0.002	1	0.02	0.003	0.04		
PEP	M+1	0	0.002	0	0	0.001	0		
PEP	M+2	0	0	0	0	0	0		
PEP	M+3	0	0.001	0	0.98	0.003	0.96		
Pyruvate	M+0	0.98	0.002	0.98	0.22	0.116	0.05	0.97	0.002
Pyruvate	M+1	0.01	0.001	0	0	0.006	0	0.02	0.001
Pyruvate	M+2	0	0	0	0	0.002	0	0	0
Pyruvate	M+3	0.02	0.002	0.01	0.77	0.119	0.95	0.01	0.002
Alanine	M+0	0.93	0.001	0.93	0.16	0.011	0.16		
Alanine	M+1	0	0.004	0	0	0.002	0		
Alanine	M+2	0.01	0.002	0.01	0	0.004	0		
Alanine	M+3	0.06	0.002	0.06	0.83	0.014	0.84		
Citrate	M+0	0.03	0.002	0.03	0.24	0.003	0.24		
Citrate	M+1	0.02	0.001	0.01	0.01	0	0.01		
Citrate	M+2	0.09	0	0.07	0.64	0.003	0.65		
Citrate	M+3	0.09	0.002	0.09	0.02	0.001	0.01		
Citrate	M+4	0.59	0.001	0.59	0.07	0.001	0.06		
Citrate	M+5	0.15	0.002	0.16	0.02	0	0.03		
Citrate	M+6	0.04	0	0.04	0	0	0		
AKG	M+0	0.04	0.003	0	0.9	0.007	0.89		
AKG	M+1	0	0.001	0.01	0	0.004	0		
AKG	M+2	0.01	0	0.01	0.09	0.002	0.1		
AKG	M+3	0.08	0.005	0.09	0.01	0.002	0.01		
AKG	M+4	0	0.003	0	0.01	0.001	0.01		
AKG	M+5	0.86	0.011	0.88	0	0	0		
Glutamine	M+0	0.02	0.001	0	1	0.001	0.99		
Glutamine	M+1	0	0	0	0	0.001	0		
Glutamine	M+2	0	0	0	0	0	0.01		
Glutamine	M+3	0	0	0.01	0	0	0		
Glutamine	M+4	0	0	0	0	0	0		
Glutamine	M+5	0.98	0	0.99	0	0	0		
Glutamate media	M+0	0.1	0.002	0	0.93	0.001	0.89		
Glutamate media	M+1	0	0	0.01	0	0	0		
Glutamate media	M+2	0.01	0	0.01	0.06	0.002	0.09		

Glutamate media	M+3	0.06	0.001	0.09	0	0	0.01
Glutamate media	M+4	0	0	0	0	0	0.01
Glutamate media	M+5	0.83	0.003	0.89	0	0	0
Glutamate	M+0	0.04	0.001	0	0.89	0.004	0.89
Glutamate	M+1	0.01	0	0.01	0	0.001	0
Glutamate	M+2	0.01	0.001	0.01	0.09	0.003	0.09
Glutamate	M+3	0.08	0.001	0.09	0.01	0	0.01
Glutamate	M+4	0	0	0	0.01	0	0.01
Glutamate	M+5	0.86	0.002	0.88	0	0	0
Fumarate	M+0	0.06	0.003	0.02	0.91	0.001	0.88
Fumarate	M+1	0.01	0.001	0.01	0.01	0.001	0.01
Fumarate	M+2	0.07	0.002	0.09	0.08	0.001	0.08
Fumarate	M+3	0.06	0.002	0.07	0	0	0.03
Fumarate	M+4	0.79	0.005	0.8	0	0.001	0
Aspartate	M+0	0.07	0.001	0.04	0.88	0.002	0.87
Aspartate	M+1	0.01	0	0.01	0.01	0.001	0.01
Aspartate	M+2	0.08	0.002	0.09	0.08	0.001	0.08
Aspartate	M+3	0.09	0.001	0.11	0.03	0.001	0.04
Aspartate	M+4	0.74	0.001	0.76	0	0	0
CarAsp	M+0	0.08	0.01	0.04	0.97	0.024	0.87
CarAsp	M+1	0	0.007	0.01	0	0.028	0.01
CarAsp	M+2	0.05	0.021	0.09	0.02	0.009	0.08
CarAsp	M+3	0.1	0.016	0.11	0.01	0.01	0.04
CarAsp	M+4	0.77	0.012	0.76	0	0	0
CarAsp	M+5	0	0	0	0	0	0
Malate	M+0	0.07	0.002	0.03	0.88	0.006	0.87
Malate	M+1	0.01	0	0.01	0.01	0.002	0.01
Malate	M+2	0.08	0.001	0.09	0.08	0.001	0.08
Malate	M+3	0.08	0.002	0.1	0.03	0.004	0.04
Malate	M+4	0.74	0.001	0.76	0	0	0
Palmitic acid	M+0	0.93	0.002		0.92	0.002	
Palmitic acid	M+1	0	0.002		0	0.001	
Palmitic acid	M+2	0.02	0.001		0.01	0	
Palmitic acid	M+3	0	0		0	0	
Palmitic acid	M+4	0.02	0.001		0.01	0	
Palmitic acid	M+5	0	0		0	0	
Palmitic acid	M+6	0.01	0		0.02	0.001	
Palmitic acid	M+7	0	0		0	0	
Palmitic acid	M+8	0.01	0		0.02	0.001	
Palmitic acid	M+9	0	0		0	0	
Palmitic acid	M+10	0.01	0		0.01	0.001	
Palmitic acid	M+11	0	0		0	0	
Palmitic acid	M+12	0	0		0.01	0.001	
Palmitic acid	M+13	0	0		0	0	
Palmitic acid	M+14	0	0		0	0	
Palmitic acid	M+15	0	0		0	0	
Palmitic acid	M+16	0	0		0	0	
Acetyl-CoA	M+0	0.65	0.02		0.05	0.004	
Acetyl-CoA	M+1	0.04	0.007		0	0.001	
Acetyl-CoA	M+2	0.27	0.012		0.07	0.004	
Acetyl-CoA	M+3	0.03	0.005		0.01	0.004	
Acetyl-CoA	M+4	0.01	0.01		0.01	0.005	

Acetyl-CoA	M+5	0	0.002	0.35	0.013
Acetyl-CoA	M+6	0	0.001	0.05	0.002
Acetyl-CoA	M+7	0	0	0.39	0.006
Acetyl-CoA	M+8	0	0	0.04	0.005
Acetyl-CoA	M+9	0	0	0.02	0.006
Acetyl-CoA	M+10	0	0	0	0
Acetyl-CoA	M+11	0	0	0	0
Acetyl-CoA	M+12	0	0	0	0
Acetyl-CoA	M+13	0	0	0	0
Acetyl-CoA	M+14	0	0	0	0
Acetyl-CoA	M+15	0	0	0	0
Acetyl-CoA	M+16	0	0	0	0
Acetyl-CoA	M+17	0	0	0	0
Acetyl-CoA	M+18	0	0	0	0
Acetyl-CoA	M+19	0	0	0	0
Acetyl-CoA	M+20	0	0	0	0
Acetyl-CoA	M+21	0	0	0	0
Acetyl-CoA	M+22	0	0	0	0
Acetyl-CoA	M+23	0	0	0	0
CoA	M+0	0.76	0.01	0.11	0.002
CoA	M+1	0.07	0.008	0.01	0.002
CoA	M+2	0.06	0.005	0.02	0.003
CoA	M+3	0	0.005	0.01	0.001
CoA	M+4	0	0	0	0.001
CoA	M+5	0.11	0.003	0.7	0.006
CoA	M+6	0	0.003	0.08	0.004
CoA	M+7	0	0	0.06	0.003
CoA	M+8	0	0.001	0.01	0.003
CoA	M+9	0	0	0	0
CoA	M+10	0	0	0	0
CoA	M+11	0	0	0	0
CoA	M+12	0	0	0	0
CoA	M+13	0	0	0	0
CoA	M+14	0	0	0	0
CoA	M+15	0	0	0	0
CoA	M+16	0	0	0	0
CoA	M+17	0	0	0	0
CoA	M+18	0	0	0	0
CoA	M+19	0	0	0	0
CoA	M+20	0	0	0	0
CoA	M+21	0	0	0	0

Supplementary Table 6. The fractional labeling of Acetyl-CoA and cytosolic Acetyl-CoA when feeding HeLa cells with [U-13C]-glucose and [U-13C]-glutamine for 24 h.

Notes:

1. Acetyl is derived from measured Acetyl-CoA and CoA (supplementary tables 2-5)
2. Cytosolic Acetyl is derived from measured Palmitic acid (tables 2-5)

Metabolite	Mass-isotopomer	Glutamine (HeLa) - derived from measurements	Glutamine (HeLa) - simulated best match	Glucose (HeLa) - derived from measurements	Glucose (HeLa) - simulated best match	Glutamine (HCT116) - derived from measurements	Glutamine (HCT116) - simulated best match	Glucose (HCT116) - derived from measurements	Glucose (HCT116) - simulated best match	Glutamine (A549) - derived from measurements	Glutamine (A549) - simulated best match	Glucose (A549) - derived from measurements	Glucose (A549) - simulated best match	Glutamine (LN229) - derived from measurements	Glutamine (LN229) - simulated best match	Glucose (LN229) - derived from measurements	Glucose (LN229) - simulated best match
Acetyl CoA	M+0	0.7	0.69	0.49	0.34	0.73	0.7	0.37	0.34	0.68	0.69	0.36	0.36	0.79	0.68	0.49	0.36
Acetyl CoA	M+1	0	0	0	0	0	0.01	0	0.01	0	0	0	0	0	0.01	0	0.01
Acetyl CoA	M+2	0.3	0.31	0.51	0.66	0.27	0.29	0.63	0.66	0.32	0.31	0.64	0.64	0.21	0.32	0.51	0.63
Acetyl CoA cytosolic	M+0	0.69	0.58	0.35	0.45	0.63	0.45	0.4	0.57	0.76	0.6	0.33	0.44	0.76	0.53	0.55	0.5
Acetyl CoA cytosolic	M+1	0	0	0	0	0	0.01	0	0.01	0	0	0	0	0	0	0	0
Acetyl CoA cytosolic	M+2	0.31	0.42	0.65	0.55	0.37	0.55	0.6	0.42	0.24	0.4	0.67	0.56	0.24	0.46	0.45	0.5

Supplementary Table 7. Metabolite measured concentrations and simulated concentrations by CODE-MFA [mM].

Metabolite	Measured concentration - HeLa (Lower bound)	Measured concentration - HeLa (Upper bound)	Simulated best match - HeLa	Measured concentration - HCT116 (Lower bound)	Measured concentration - HCT116 (Upper bound)	Simulated best match - HCT116	Measured concentration - A549 (Lower bound)	Measured concentration - A549 (Upper bound)	Simulated best match - A549	Measured concentration - LN229 (Lower bound)	Measured concentration - LN229 (Upper bound)	Simulated best match - LN229
13BPG	0.00067	0.0033	0.00082	0.0015	0.0023	0.0015	0.0003	0.0011	0.00061	0.001	0.0039	0.0011
Acetyl_CoA	0.001	0.005	0.003	0.00024	0.0035	0.0019	0.0072	0.011	0.009	0.007	0.011	0.0089
CoA	0.0054	0.0094	0.0074	0.0011	0.0017	0.0014	0.018	0.027	0.023	0.017	0.025	0.021
6phosphogluconate	0.011	0.024	0.011	0.0071	0.018	0.0071	0.0016	0.0065	0.0046	0.037	0.12	0.037
NADP	0.012	0.028	0.02	0.0057	0.017	0.012	0.11	0.14	0.12	0.037	0.057	0.047
GDP	0.002	0.03	0.016	0.00001	0.017	0.0052	0.0074	0.011	0.0091	0.015	0.046	0.031
AKG	0.022	0.038	0.031	0.049	0.12	0.083	0.012	0.021	0.017	0.013	0.03	0.024
OAA	0.04	0.06	0.048	0.00001	0.1	0.05	0.00001	0.1	0.049	0.00001	0.1	0.044
Glc6P	0.022	0.071	0.03	0.026	0.18	0.096	0.0067	0.019	0.0077	0.017	0.15	0.054
Fumarate	0.033	0.097	0.068	0.03	0.11	0.1	0.035	0.056	0.046	0.12	0.15	0.13
NADH	0.073	0.11	0.082	0.021	0.032	0.026	0.41	0.61	0.43	0.024	0.037	0.03
NADPH	0.092	0.14	0.116	0.0067	0.015	0.011	0.31	0.72	0.52	0.036	0.053	0.045
Pyruvate	0.08	0.16	0.112	0.087	0.15	0.12	0.084	0.12	0.093	0.058	0.25	0.16
3PG	0.13	0.17	0.13	0.00001	0.543	0.000017	0.082	0.13	0.13	0.031	0.62	0.067
PEP	0.024	0.22	0.03	0.016	0.038	0.027	0.016	0.027	0.022	0.015	0.1	0.057
Citrate	0.15	0.24	0.19	0.39	1.096	0.74	0.016	0.26	0.14	0.12	0.47	0.3
R5P	0.21	0.33	0.33	0.084	0.28	0.11	0.069	0.15	0.07	0.29	0.5	0.5
Malate	0.27	0.41	0.35	0.1	0.65	0.26	0.15	0.31	0.25	0.28	0.4	0.36
Lactate	0.28	0.44	0.39	0.29	0.46	0.37	0.37	0.56	0.51	0.19	0.7	0.42
Alanine	0.28	0.51	0.4	0.53	0.58	0.55	0.025	0.04	0.033	0.17	0.31	0.24
ADP	0.28	0.52	0.4	0.00001	0.33	0.23	0.21	0.31	0.26	0.26	1.49	0.88
NAD	0.4	0.56	0.54	0.34	0.97	0.66	1.14	1.52	1.55	0.91	1	0.96
G3P	0.49	0.65	0.54	0.22	0.98	0.98	0.21	0.36	0.32	0.034	0.68	0.58
GTP	0.54	0.7	0.62	0.00001	1.43	0.59	0.34	0.49	0.42	0.00001	4.04	1.9
Aspartate	1.11	1.75	1.48	0.65	0.83	0.74	0.85	1.2	1.05	5.59	7.73	7.37
ATP	0.89	3.97	2.43	1.77	4.93	2.33	0.53	1.35	0.94	1.23	7.98	4.61
Orthophosphate	5.43	6.23	5.83	0.00001	11.66	5.83	0.00001	11.66	5.83	0.00001	11.66	5.83
Glutamate	9.4	14.6	10.19	9.23	13.43	11.37	9.15	13.09	10.048	16.88	22.82	10.83
Glutathione	8.53	15.27	11.9	11.2	17.32	14.26	8.28	13.54	10.91	9.03	13.1	11.07
Glutamine	11.64	15.56	13.6	39.43	54.87	47.15	2.22	3.27	2.74	8.46	12.29	10.37

Supplementary Table 8. Metabolite concentrations derived by CODE-MFA [mM].

met name	Simulated concentration - HeLa (Lower bound)	Simulated concentration - HeLa (Upper bound)	Simulated best match - HeLa	Simulated concentration - HCT116 (Lower bound)	Simulated concentration - HCT116 (Upper bound)	Simulated best match - HCT116	Simulated concentration - A549 (Lower bound)	Simulated concentration - A549 (Upper bound)	Simulated best match - A549	Simulated concentration - LN229 (Lower bound)	Simulated concentration - LN229 (Upper bound)	Simulated best match - LN229
Glutamine_CY	0.0021	19.45	16.98	18.27	50	50	0.0009	4.08	0.0025	0.0031	15.36	0.47
Glutamine_MT	0.0016	50	0.088	0.0017	50	37.06	0.00023	16.32	13.7	0.00083	50	50
ADP_MT	0.000035	2.11	0.28	0.00001	0.24	0.000029	0.00002	1.57	0.058	0.000035	5.76	2.12
ADP_CY	0.158	0.65	0.43	0.11	0.42	0.29	0.015	0.39	0.31	0.21	1.53	0.56
Orthophosphate_CY	0.0019	7.79	2.08	0.0021	14.57	0.0057	0.0015	12.16	1.64	0.03	12.2	4.49
Orthophosphate_MT	0.00035	31.15	20.83	0.00049	50	29.13	0.00038	50	22.57	0.00051	50	11.2
Glutamate_CY	0.086	0.63	0.23	5.21	16.78	14.18	0.003	0.16	0.06	0.14	2.83	1.04
ATP_MT	0.000016	3.9	0.35	0.00001	0.35	0.000044	0.00001	2.36	0.029	0.000017	7.78	2.86
ATP_CY	1.085	4.96	2.95	1.073	4.19	2.92	0.16	1.69	1.17	1.85	9.97	5
NH3_MT	0.00001	50	50	0.00001	50	0.0005	0.00001	50	0.000018	0.00001	50	0.018
Glutamate_MT	18.39	50	50	0.0067	7.31	0.13	18.4	50	50	18.4	50	50
NAD_MT	0.53	2.8	1.44	0.052	4.84	2.85	1.37	7.58	3.72	0.071	5	3.87
NADH_MT	0.151	0.56	0.41	0.048	0.16	0.13	0.8	3.05	2.17	0.056	0.18	0.15
NADP_MT	0.00001	0.000027	0.00001	0.00001	0.0015	0.00001	0.00001	0.0023	0.000015	0.00001	0.011	0.00001
NADPH_MT	0.213	0.7	0.58	0.02	0.076	0.053	0.94	3.58	2.55	0.081	0.27	0.22
NAD_CY	0.118	0.7	0.32	0.04	1.21	0.11	0.37	1.89	1.01	0.031	1.25	0.23
NADH_CY	0.000015	0.00011	0.000041	0.00001	0.00026	0.000013	0.00008	0.0006	0.00022	0.00001	0.00031	0.000015
NADP_CY	0.0092	0.035	0.025	0.0053	0.022	0.014	0.056	0.17	0.15	0.022	0.072	0.059
NADPH_CY	0.0003	0.006	0.0008	0.000063	0.0034	0.00047	0.00028	0.015	0.0056	0.000041	0.016	0.00082
Acetyl_CoA_MT	0.00008	0.012	0.0043	0.000035	0.014	0.0052	0.00008	0.032	0.012	0.00004	0.044	0.016
OAA_MT	0.00001	0.000027	0.00001	0.00001	0.0014	0.00007	0.00001	0.000027	0.00001	0.00001	0.00068	0.000012
Citrate_MT	0.355	1.18	0.96	1.36	5.48	3.69	0.25	1.3	0.69	0.54	2.37	1.48
CoA_MT	0.00001	0.047	0.0088	0.00001	0.0085	0.00001	0.00001	0.14	0.02	0.00001	0.12	0.0063
CoA_CY	0.0009	0.012	0.007	0.00001	0.0021	0.0018	0.0032	0.034	0.023	0.00001	0.031	0.024
Fumarate_MT	0.036	0.26	0.097	0.008	0.56	0.44	0.074	0.28	0.2	0.14	0.76	0.38
Malate_MT	0.094	0.69	0.25	0.021	1.45	1.15	0.19	0.73	0.52	0.016	2	0.044
Pyruvate_MT	0.00001	0.00038	0.000019	0.0002	0.04	0.03	0.00001	0.031	0.023	0.00001	0.003	0.00002
PEP_MT	0.00034	0.0069	0.0025	0.00001	0.19	0.004	0.00001	0.0015	0.00001	0.00001	0.0015	0.00001
GDP_MT	0.00057	0.084	0.031	0.00001	0.068	0.025	0.00001	0.053	0.041	0.00001	0.23	0.14
GTP_MT	0.174	3.48	1.29	0.00003	4.7	0.23	0.000016	2.45	0.0066	0.00012	20.1	0.018
GDP_CY	0.00001	0.033	0.012	0.00001	0.022	0.00016	0.00001	0.013	0.0012	0.00001	0.054	0.0027
GTP_CY	0.00015	0.87	0.45	0.000084	1.78	0.68	0.19	0.61	0.52	0.0003	5	2.37
PEP_CY	0.013	0.1	0.037	0.000011	0.047	0.032	0.01	0.034	0.027	0.026	0.12	0.071
Pyruvate_CY	0.051	0.2	0.14	0.052	0.19	0.14	0.041	0.15	0.11	0.073	0.31	0.2
AKG_MT	0.00013	0.0027	0.00037	0.00021	0.084	0.00056	0.000014	0.002	0.00074	0.00024	0.1	0.00066
Aspartate_MT	0.19	1.4	0.52	0.000027	1.59	0.00053	0.43	6.02	1.18	0.22	33.3	0.61
Aspartate_CY	0.63	2.19	1.72	0.12	1.04	0.93	0.14	1.51	1.02	3.33	9.66	9
OAA_CY	0.022	0.075	0.06	0.023	0.12	0.062	0.022	0.12	0.061	0.02	0.12	0.054
Citrate_CY	0.000039	0.00079	0.0003	0.00014	0.02	0.00037	0.00002	0.0011	0.0004	0.000045	0.0067	0.00033
AKG_CY	0.014	0.047	0.039	0.038	0.14	0.1	0.0078	0.026	0.021	0.011	0.037	0.03
Malate_CY	0.136	0.51	0.37	0.0058	0.38	0.043	0.065	0.39	0.18	0.06	0.51	0.44
Fumarate_CY	0.022	0.084	0.061	0.0021	0.14	0.016	0.00001	0.057	0.0077	0.000024	0.19	0.072
Acetyl_CoA_CY	0.00036	0.0062	0.0027	0.00001	0.0028	0.001	0.0031	0.014	0.0084	0.0026	0.013	0.0071
Glutathione_MT	0.0004	50	0.022	0.00073	50	16	0.00083	50	50	0.00096	50	0.0026
Glutathione_CY	2.012	19.1	14.87	0.69	21.65	13.8	0.0028	16.9	1.14	0.0017	16.38	13.8
Lactate_CY	0.18	0.55	0.48	0.17	0.58	0.47	0.23	0.7	0.64	0.2	0.87	0.53
Alanine_CY	0.000074	0.6	0.0002	0.00017	0.72	0.5	0.00001	0.05	0.029	0.000044	0.35	0.00012
Alanine_MT	0.272	2.57	2	0.28	2.9	0.76	0.017	0.2	0.045	0.16	1.54	1.19
Glc6P_CY	0.022	0.07	0.03	0.026	0.18	0.1	0.0066	0.019	0.0077	0.017	0.15	0.054
R5P_CY	0.21	0.33	0.33	0.083	0.28	0.11	0.069	0.15	0.07	0.29	0.5	0.5
CarAsp_CY	0.00001	50	0.00023	0.00001	50	0.000023	0.00001	50	0.0067	0.00001	50	0.018
Asparagine_CY	0.00001	50	0.0021	0.00001	50	0.12	0.00001	50	0.00026	0.00001	50	0.00045
3PG_CY	0.13	0.17	0.13	0.00001	0.36	0.000016	0.082	0.13	0.13	0.031	0.62	0.067
13BPG_CY	0.00068	0.0033	0.00082	0.0014	0.0023	0.0014	0.0003	0.0011	0.00061	0.001	0.0039	0.0011
G3P_CY	0.49	0.65	0.54	0.22	0.98	0.98	0.21	0.36	0.32	0.034	0.68	0.58
6phosphogluconate_CY	0.011	0.024	0.011	0.0071	0.018	0.0071	0.0016	0.0065	0.0046	0.037	0.12	0.037

Supplementary Table 9. Uptake/secretion rate measurements [mM/h]

Reaction #	Reaction desctiption	Measured flux - HeLa (lb)	Measured flux - HeLa (ub)	Measured flux - HCT116 (lb)	Measured flux - HCT116 (ub)	Measured flux - A549 (lb)	Measured flux - A549 (ub)	Measured flux - LN229 (lb)	Measured flux - LN229 (ub)
v1	Glutamine_Media => Glutamine_CY	81	82	56	58	53	54.6	36	38
v2	Glutamate_CY => Glutamate_Media	0.1	70	0.1	24.8	0.1	15.6	0.1	19.7
v3	Glucose_Media => Glc6P_CY	111	355	214	272	188	232	133	294
v4	Lactate_CY => Lactate_Media	506	643	476	612	398	481	414	465
v5	Pyruvate_CY => Pyruvate_Media	3.5	4.8	26	31	13.5	17.8	20.5	25.4

Supplementary Table 10. Net fluxes derived by CoDe-MFA [mM/h]

Reaction #	Reaction desctiption	Flux by CODE-MFA - HeLa (lb)	Flux by CODE-MFA - HeLa (ub)	Flux by CODE-MFA - HeLa (best fit)	Flux by CODE-MFA - HCT116 (lb)	Flux by CODE-MFA - HCT116 (ub)	Flux by CODE-MFA - HCT116 (best fit)	Flux by CODE-MFA - A549 (lb)	Flux by CODE-MFA - A549 (ub)	Flux by CODE-MFA - A549 (best fit)	Flux by CODE-MFA - LN229 (lb)	Flux by CODE-MFA - LN229 (ub)	Flux by CODE-MFA - LN229 (best fit)
v1	Glutamine_Media => Glutamine_CY	81	82	82	56	58	58	53	54.6	54.6	36	38	38
v2	Glutamate_CY => Glutamate_Media	53.79	70	63.03	4.92	24.14	14.92	13.6	15.6	15.6	0.79	19.7	10.79
v3	Glucose_Media => Glc6P_CY	256.02	263.27	257.27	231	272	272	199	232	232	206.31	246.31	239.31
v4	Lactate_CY => Lactate_Media	506	516.5	506	495.11	584.15	565.15	407.09	481	477.09	414	465	465
v5	Pyruvate_CY => Pyruvate_Media	3.5	4.8	4.8	26	31	26	13.5	17.8	17.8	20.5	25.4	25.4
v13	Aspartate_CY => Fumarate_CY	2	4	2.97	2	4	2	2	4	4	2	4	2
v16	R5P_CY => R5P_CY_Pool	2	4	2	2	4	2	2	4	2	2	4	2
v17	Acetyl_CoA_CY => Fatty_Acid	1.19	5.19	2.69	5	10	10	6.5	10	10	0.13	3.63	1.13
v18	CarAsp_CY => Pyrimidines	2	4	2	2	4	2	2	4	4	2	4	4
v19	Asparagine_CY => Asparagine_CY_Pool	1	3	1	1	3	1	1	3	3	1	3	3
v21	Proline_MT => Proline_MT_Pool	1	3	3	1	3	3	1	3	3	1	3	3
v51	Glutamine_CY => Glutamine_MT	38	82	79	22.01	58	55.01	0	54.6	38.6	0	38	4.72
v52.1	Glutamate_CY => Glutamate_MT	0	25	0	-55.67	10.33	-14.67	-202.35	100.4	-44.27	-90	96	0
v53.1	AKG_CY + Malate_MT => AKG_MT + Malate_CY	0.9	14.4	4.4	0	18.41	4.41	10.63	74.63	24.63	-3.5	100	0
v53.2	AKG_MT + Glutathione_CY => AKG_CY + Glutathione_MT	52.29	100.61	75.61	0	38.56	13.56	-140	90	0	-88.75	111.25	11.25
v55	Fumarate_MT => Fumarate_CY	-14	-2	-2.97	-4	29.48	17.48	-55.66	-2	-14.66	-96.21	-2.21	-16.21
v56.1	Malate_MT + Citrate_CY => Malate_CY + Citrate_MT	-4.36	0	-1.36	-9.56	-0.87	-5.87	-12.01	-7.51	-11.01	-8.29	-0.29	-5.29
v57	Aspartate_MT + Glutamate_CY => Aspartate_CY + Glutamate_MT	5.35	17.82	7.82	3.96	11.96	4.96	9.07	22.57	19.07	19.67	47.67	38.67
v58	Alanine_MT => Alanine_CY	0	3	0.36	1.1	7.49	2.49	3.5	10	6.57	4.3	10.8	4.8
v59	Pyruvate_MT => Pyruvate_CY	-9.84	-3.84	-6.34	-16.74	-8.24	-11.74	-20.45	-12.45	-18.45	-14.93	-8.93	-9.93
v60	PEP_CY + Citrate_MT => PEP_MT + Citrate_CY	0	2.98	0.98	0	1.19	0.19	0.02	1.52	1.02	0	1.28	0.78
v101	Glutamine_CY => Glutamine_CY_Pool	0	3	3	0	3	2.99	0	3	0	0	3	0
v102	Glutamine_CY => Glutamate_CY	0	41	0	0	33	0	0	53.6	16	0	37	33.28
v105	Glutamate_CY => Glutamate_CY_Pool	0	3	0	0	3	0	0	3	3	0	3	0
v106.1	Glutamate_CY => AKG_CY	-88	-50	-69	-24.25	26.35	-5.25	-70.25	169.75	29.75	-69.51	100.49	10.49
v107	Citrate_CY + NADP_CY => AKG_CY + NADPH_CY + CO2	-1.36	0	-0.36	-7.94	-0.44	-3.94	1.03	3.53	2.03	0	6.43	4.93
v108	Citrate_CY + ATP_CY + CoA_CY => Acetyl_CoA_CY + OAA_CY + ADP_CY + Orthophosphate_CY	1.19	5.19	2.69	5	10	10	6.5	10	10	0.13	3.63	1.13
v109	Fumarate_CY => Malate_CY	-10	0	0	0	15.74	9.74	-30.33	0	-5.33	-57.1	-0.1	-7.1
v111	Malate_CY + NAD_CY => OAA_CY + NADH_CY	-8.54	-2.04	-4.54	-10	-3.96	-9.96	-18.59	-10.16	-17.16	-34.8	-13.8	-27.8
v112	Malate_CY + NADP_CY => Pyruvate_CY + CO2 + NADPH_CY	2.59	10.09	7.59	24.97	33.97	27.97	18.61	23.11	20.11	0	18.31	8.31
v113	OAA_CY + Glutamate_CY => Aspartate_CY + AKG_CY	-5.35	-0.35	-1.85	0	1.04	0.04	-8.66	-0.16	-7.16	-33.67	-12.67	-26.67
v114	Aspartate_CY + CO2 => CarAsp_CY	2	4	2	2	4	2	2	4	4	2	4	4
v115	Aspartate_CY => Asparagine_CY	1	3	1	1	3	1	1	3	3	1	3	3
v116	Aspartate_CY => Aspartate_CY_Pool	0	3	0	0	3	0	0	3	0.92	0	3	3
v117	Alanine_CY => Alanine_CY_Pool	0	3	0.36	0	3	1	0	3	0	0	3	0
v118	Pyruvate_CY + NADH_CY => Lactate_CY + NAD_CY	506	516.5	506	495.11	584.15	565.15	407.09	481	477.09	414	465	465
v119	PEP_CY + ADP_CY => Pyruvate_CY + ATP_CY	506.56	518.54	509.56	504.92	593.92	574.92	423.23	498.23	493.23	426.52	499.02	492.02
v120	3PG_CY => PEP_CY	508.04	518.54	510.54	505.11	594.11	575.11	423.24	499.24	494.24	427.8	499.8	492.8
v121	13BPG_CY + ADP_CY => 3PG_CY + ATP_CY	508.04	518.54	510.54	505.11	594.11	575.11	423.24	499.24	494.24	427.8	499.8	492.8
v122	G3P_CY + NAD_CY + Orthophosphate_CY => 13BPG_CY + NADH_CY	508.04	518.54	510.54	505.11	594.11	575.11	423.24	499.24	494.24	427.8	499.8	492.8
v123	Glc6P_CY => G3P_CY + G3P_CY	254.02	259.27	255.27	252.55	297.05	287.55	211.62	249.62	247.12	213.9	249.9	246.4
v124	Glc6P_CY + NADP_CY => 6phosphogluconate_CY + NADPH_CY	2	4	2	2	4	2	2	4	2	2	4	2
v125	6phosphogluconate_CY + NADP_CY => R5P_CY + NADPH_CY + CO2	2	4	2	2	4	2	2	4	2	2	4	2
v201	Glutamine_MT => Glutamine_MT_Pool	0	3	0	0	3	0.01	0	3	3	0	3	3
v202	Glutamine_MT => Glutamate_MT + NH3	38	81	79	22	57	55	0	53.6	35.6	0	37	1.72
v203	Glutamate_MT => Proline_MT	1	3	3	1	3	3	1	3	3	1	3	3
v204	Glutamate_MT => Glutamate_MT_Pool	0	3	3	0	3	3	0	3	0	0	3	3
v205.1	Glutamate_MT => AKG_MT	44.99	93.32	69.99	-2.16	49.84	30.84	-163.75	86.22	-23.32	-109.3	80.42	-9.58
v206	AKG_MT + CO2 + NADPH_MT => Citrate_MT + NADP_MT	0.12	1.12	0.62	-7.98	-0.48	-5.48	0.74	3.24	2.24	0.26	1.76	0.76
v207	AKG_MT + CO2 + NADH_MT => Citrate_MT + NAD_MT	-2.98	-0.98	-1.98	-10	0	0	-1.5	0	0	-6	0	0
v208	Acetyl_CoA_MT + OAA_MT => Citrate_MT + CoA_MT	2.2	6.2	3.7	8.04	15.54	11.54	6.79	10.79	9.79	4.31	7.81	5.31
v209	AKG_MT => CO2 + Fumarate_MT	6.98	20.98	10.98	29.62	45.62	35.62	29.29	31.79	29.79	12.38	32.38	22.38
v210	Fumarate_MT => Malate_MT	10.95	25.95	6.97	4.07	28.07	9.07	15.22	47.22	22.22	9.29	69.29	19.29
v212	Malate_MT + NAD_MT => OAA_MT + NADH_MT	7.9	20.9	10.9	13.81	27.31	17.31	19.93	31.93	29.93	24.19	53.19	43.19
v213.1	Malate_MT + NAD_MT => Pyruvate_MT + CO2 + NADH_MT	0	0.2	0	0.29	3.29	2.29	0	2.41	0.91	0.18	1.18	0.68
v213.2	Malate_MT + NADP_MT => Pyruvate_MT + CO2 + NADPH_MT	-0.5	0	0	0	0	0	-1	1	0	0	0	0
v214	Aspartate_MT + AKG_MT => OAA_MT + Glutamate_MT	-18.81	-6.35	-8.81	-14	-4.96	-5.96	-22.66	-11.16	-21.16	-47.67	-19.67	-38.67
v215	Aspartate_MT => Aspartate_MT_Pool	0	3	1	0	3	1	0	3	2.08	0	3	0
v216	Alanine_MT => Alanine_MT_Pool	0	3	2.15	0	3	0	0	3	3	0	3	1
v217	Pyruvate_MT + Glutamate_MT => Alanine_MT + AKG_MT	0.51	3	2.01	2.1	7.49	2.49	4.5	10	9.56	5.3	10.3	5.3
v218	PEP_MT + CO2 + GDP_MT => OAA_MT + GTP_MT	0	2.98	0.98	0	1.19	0.19	0.02	1.52	1.02	0	1.28	0.78
v219	Pyruvate_MT + NAD_MT + CoA_MT => CO2 + Acetyl_CoA_MT + NADH_MT	2.2	6.2	3.7	8.04	15.54	11.54	6.79	10.79	9.79	4.31	7.81	5.31
v220	Pyruvate_MT + CO2 + ATP_MT => OAA_MT + Orthophosphate_MT + ADP_MT	0	1.63	0.63	0	1	0	0	1	0	0	1	0

Supplementary Table 11. Thermodynamic calculations for mitochondrial transporters

Constants	Value	Units
RT	2.478	
F (Faraday constant)	96.49	kC/mol
Mitochondrial PH level	7.5	pH
Cytosolic PH level	7	pH
φ (Mitochondrial membrane potential)	0.15	V

Mitochondrial transport

aKG[m] + Glutathione[c] => aKG[c] + Glutathione[m]

Standard gibbs free energy formula

G0(aKG[c]) + G0(Glutathione[m]) - G0(aKG[m]) - G0(Glutathione[c]) - numOfProtons*RT(ln(10^(-mitochondrial PH))-ln(10^(-Cytisolic PH))) - Fφ(-z(aKG[m])+z(Glutathione[c]))

Gibbs free enrgy of formation	Value	Units
aKG[m] formation (in pH 7.5)	-619.7	kJ/mol
aKG[c] formation (in pH 7.0)	-631.1	kJ/mol
Glutathione[m] formation (in pH 7.5)	-242.2	kJ/mol
Glutathione[c] formation (in pH 7.0)	-287.9	kJ/mol

Charge (z) - based on most abundant species	Value
z(aKG[m])	-2
z(Glutathione[c])	-1

number of protons/hydrogens - based on most abundant species	Value
aKG	4
Glutathione	16

Standard gibbs free energy of transporter	Value	Units
	-14.4083	kJ/mol

Mitochondrial transport

Ala[m] =>Ala[c]

Standard gibbs free energy formula

G0(Ala[c]) - G0(Ala[m]) - numOfProtons*RT(ln(10^(-mitochondrial PH))-ln(10^(-Cytisolic PH))) - Fφ(-z(Ala[m]))

Gibbs free enrgy of formation	Value	Units
Ala[m] formation (in pH 7.5)	-62.9	kJ/mol
Ala[c] formation (in pH 7.0)	-82.8	kJ/mol

Charge (z) - based on most abundant species	Value
z(Ala[m])	0

number of protons/hydrogens - based on most abunda	Value
Ala	7

Standard gibbs free energy of transporter	Value	Units
	0.070321	kJ/mol

Mitochondrial transport

Cit[m] + PEP[c] => Cit[c] + PEP[m]

Standard gibbs free energy formula

G0(Cit[c]) + G0(PEP[m]) - G0(Cit[m]) - G0(PEP[c]) - numOfProtons*RT(ln(10^(-mitochondrial PH))-ln(10^(-Cytisolic PH))) - Fφ(-z(Cit[m])+ z(PEP[c]))

Gibbs free enrgy of formation	Value	Units
Cit[m] formation (in pH 7.5)	-951.7	kJ/mol
Cit[c] formation (in pH 7.0)	-966	kJ/mol
PEP[m] formation (in pH 7.5)	-1184.6	kJ/mol
PEP[c] formation (in pH 7.0)	-1190.3	kJ/mol

Charge (z) - based on most abundant species	Value
z(Cit[m])	-3
z(PEP[c])	-3

number of protons/hydrogens - based on most abund	Value
Cit	5
PEP	2

Standard gibbs free energy of transporter	Value	Units
	-0.041291209	kJ/mol

Mitochondrial transport

Mal[m] + aKG[c] => Mal[c] + aKG[m]

Standard gibbs free energy formula

G0(Mal[c]) + G0(aKG[m]) - G0(Mal[m]) - G0(aKG[c]) - numOfProtons*RT(ln(10^(-mitochondrial PH))-ln(10^(-Cytisolic PH))) - Fφ(-z(Mal[m])+ z(aKG[c]))

Gibbs free enrgy of formation	Value	Units
Mal[m] formation (in pH 7.5)	-669.3	kJ/mol
Mal[c] formation (in pH 7.0)	-680.7	kJ/mol
aKG[m] formation (in pH 7.5)	-619.7	kJ/mol
aKG[c] formation (in pH 7.0)	-631.1	kJ/mol

Charge (z) - based on most abundant species	Value
z(Mal[m])	-2
z(aKG[c])	-2

number of protons/hydrogens - based on most abundan	Value
Mal	4
aKG	4

Standard gibbs free energy of transporter	Value	Units
	-1.13687E-13	kJ/mol

Mitochondrial transport

Mal[m] + Cit[c] => Mal[c] + Cit[m]

Standard gibbs free energy formula

G0(Mal[c]) + G0(Cit[m]) - G0(Mal[m]) - G0(Cit[c]) - numOfProtons*RT(ln(10^(-mitochondrial PH))-ln(10^(-Cytisolic PH))) - Fφ(-z(Mal[m])+ z(Cit[c]))

Gibbs free enrgy of formation	Value	Units
Mal[m] formation (in pH 7.5)	-669.3	kJ/mol
Mal[c] formation (in pH 7.0)	-680.7	kJ/mol
Cit[m] formation (in pH 7.5)	-951.7	kJ/mol
Cit[c] formation (in pH 7.0)	-966	kJ/mol

Charge (z) - based on most abundant species	Value
z(Mal[m])	-2
z(Cit[c])	-3

number of protons/hydrogens - based on most abundant s	Value
Mal	4
Cit	5

Standard gibbs free energy of transporter	Value	Units
	14.52059707	kJ/mol

Mitochondrial transport

Asp[m] + Glu[c] + h[c] => Asp[c] + Glu[m] + h[m]

Standard gibbs free energy formula

G0(Asp[c]) + G0(Glu[m]) - G0(Asp[m]) - G0(Glu[c]) - numOfProtons*RT(ln(10^(-mitochondrial PH))-ln(10^(-Cytisolic PH))) - Fφ(-z(Asp[m])+z(Glu[c])+z(H+[c]))

Gibbs free enrgy of formation	Value	Units
Asp[m] formation (in pH 7.5)	-437.5	kJ/mol
Asp[c] formation (in pH 7.0)	-454.6	kJ/mol
Glu[m] formation (in pH 7.5)	-345.6	kJ/mol
Glu[c] formation (in pH 7.0)	-368.3	kJ/mol

Charge (z) - based on most abundant species	Value
z(Asp[m])	-1
z(Glu[c])	-1
z(H+[c])	1

number of protons/hydrogens - based on most abundant sp	Value
Asp	6
Glu	8
H+	1

Standard gibbs free energy of transporter	Value	Units
	-17.4322	kJ/mol

Mitochondrial transport

Pyr[m] + h[m] => Pyr[c] + h[c]

Standard gibbs free energy formula

G'0(Pyr[c]) - G'0(Pyr[m]) - numOfProtons*RT(ln(10^(-mitochondrial PH))-ln(10^(-Cytisolic PH))) - Fφ(-z(Pyr[m])- z(H+[m]))

Gibbs free enrgy of formation	Value	Units
Pyr[m] formation (in pH 7.5)	-337.3	kJ/mol
Pyr[c] formation (in pH 7.0)	-345.8	kJ/mol

Charge (z) - based on most abundant species	Value
z(Pyr[m])	-1
z(H+[m])	1

number of protons/hydrogens - based on most abun	Value
Pyr	3
H+	1

Standard gibbs free energy of transporter	Value	Units
	2.911612	kJ/mol

Supplementary Table 12. Previously published co-factor rations in cytosol and mitochondria

NAD+/NADH [c]	Reference
6.7	Chen, W. W., Freinkman, E., Wang, T., Birsoy, K., & Sabatini, D. M. (2016). Absolute Quantification of Matrix Metabolites Reveals the Dynamics of Mitochondrial Metabolism. Cell. https://doi.org/10.1016/j.cell.2016.07.040
725	Krebs, H. A. (1967). The redox state of nicotinamide adenine dinucleotide in the cytoplasm and mitochondria of rat liver. Advances in Enzyme Regulation. https://doi.org/10.1016/0065-2571(67)90029-5
88	Sun, F., Dai, C., Xie, J., & Hu, X. (2012). Biochemical issues in estimation of cytosolic free NAD/NADH ratio. PLoS ONE. https://doi.org/10.1371/journal.pone.0034525
1000	Hedeskov, C. J., Capito, K., & Thams, P. (1987). Cytosolic ratios of free [NADPH]/[NADP+] and [NADH]/[NAD+] in mouse pancreatic islets, and nutrient-induced insulin secretion. The Biochemical Journal, 241(1), 161–167. https://doi.org/10.1042/bj2410161
800	Zhao, Y., Hu, Q., Cheng, F., Su, N., Wang, A., Zou, Y., … Yang, Y. (2015). SoNar, a Highly Responsive NAD+/NADH Sensor, Allows High-Throughput Metabolic Screening of Anti-tumor Agents. Cell Metabolism. https://doi.org/10.1016/j.cmet.2015.04.009

NAD+/NADH [m]	Reference
67	Lee, W. D., Mukha, D., Aizenshtein, E., & Shlomi, T. (2019). Spatial-fluxomics provides a subcellular-compartmentalized view of reductive glutamine metabolism in cancer cells. Nature Communications. https://doi.org/10.1038/s41467-019-09352-1
111	Chen, W. W., Freinkman, E., Wang, T., Birsoy, K., & Sabatini, D. M. (2016). Absolute Quantification of Matrix Metabolites Reveals the Dynamics of Mitochondrial Metabolism. Cell. https://doi.org/10.1016/j.cell.2016.07.040
8	Krebs, H. A. (1967). The redox state of nicotinamide adenine dinucleotide in the cytoplasm and mitochondria of rat liver. Advances in Enzyme Regulation. https://doi.org/10.1016/0065-2571(67)90029-5
60	Siess, E. A., Brocks, D. G., Lattke, H. K., & Wieland, O. H. (1977). Effect of glucagon on metabolite compartmentation in isolated rat liver cells during gluconeogenesis from lactate. Biochemical Journal. https://doi.org/10.1042/bj1660225

NADP+/NADPH [c]	Reference
0.03	Hedeskov, C. J., Capito, K., & Thams, P. (1987). Cytosolic ratios of free [NADPH]/[NADP+] and [NADH]/[NAD+] in mouse pancreatic islets, and nutrient-induced insulin secretion. The Biochemical Journal, 241(1), 161–167. https://doi.org/10.1042/bj2410161
0.01	Veech, R. L., Eggleston, L. V, & Krebs, H. a. (1969). The redox state of free nicotinamide-adenine dinucleotide phosphate in the cytoplasm of rat liver. The Biochemical Journal, 115(4), 609–619. https://doi.org/10.1042/bj1150609a
0.038	Siess, E. A., Brocks, D. G., Lattke, H. K., & Wieland, O. H. (1977). Effect of glucagon on metabolite compartmentation in isolated rat liver cells during gluconeogenesis from lactate. Biochemical Journal. https://doi.org/10.1042/bj1660225
0.0133	Sallin, O., Reymond, L., Gondrand, C., Raith, F., Koch, B., & Johnsson, K. (2018). Semisynthetic biosensors for mapping cellular concentrations of nicotinamide adenine dinucleotides. ELife. https://doi.org/10.7554/eLife.32638

NADP+/NADPH [m]	Reference
0.012	SIES, H., AKERBOOM, T. P. M., & TAGER, J. M. (1977). Mitochondrial and Cytosolic NADPH Systems and Isocitrate Dehydrogenase Indicator Metabolites during Ureogenesis from Ammonia in Isolated Rat Hepatocytes. European Journal of Biochemistry. https://doi.org/10.1111/j.1432-1033.1977.tb11253.x
0.005	Sallin, O., Reymond, L., Gondrand, C., Raith, F., Koch, B., & Johnsson, K. (2018). Semisynthetic biosensors for mapping cellular concentrations of nicotinamide adenine dinucleotides. ELife. https://doi.org/10.7554/eLife.32638

Supplementary Table 13. Metabolite measured concentrations for 24 h and 48 h incubation period in HeLa cells [mM].

Metabolite	Measured concentration - 24 hours (Lower bound)	Measured concentration - 24 hours (Upper bound)	Measured concentration - 48 hours (Lower bound)	Measured concentration - 48 hours (Upper bound)
13BPG	0.00067	0.0033	0.0013	0.0017
Glc6P	0.022	0.071	0.015	0.07
Fumarate	0.033	0.097	0.089	0.12
3PG	0.13	0.17	0.15	0.16
Citrate	0.15	0.24	0.18	0.27
R5P	0.21	0.33	0.16	0.39
Malate	0.27	0.41	0.44	0.61
Lactate	0.28	0.44	0.44	0.8
Alanine	0.28	0.51	0.51	0.68
ADP	0.28	0.52	0.5	0.53
G3P	0.49	0.65	0.9	1.24
GTP	0.54	0.7	0.84	1.1
Aspartate	1.11	1.75	1.97	2.53
ATP	0.89	3.97	3.04	3.24
Glutamate	9.4	14.6	12.94	14.83
Glutathione	8.53	15.27	10.6	11.4
Glutamine	11.64	15.56	14.17	14.63

Supplementary Table 14. The fractional labeling of metabolites when feeding HeLa cells with [U-13C]-glucose and [U-13C]-glutamine for 24 h and 48 h, using pHilic method.

Metabolite	Mass-isotopomer	Glutamine 24h (measured mean - raw data)	Glutamine 24h (measured SD)	Glutamine 48h (measured mean - raw data)	Glutamine 48h (measured SD)	Glucose 24h (measured mean - raw data)	Glucose 24h (measured SD)	Glucose - 48 (measured mean - raw data)	Glucose 48h (measured SD)
Lactate	M+0	0.96	0.001	0.96	0	0.04	0.002	0.06	0.04
Lactate	M+1	0.03	0.001	0.03	0	0	0	0	0.001
Lactate	M+2	0	0	0	0	0.03	0.002	0.03	0.003
Lactate	M+3	0.01	0	0.01	0	0.93	0.001	0.91	0.04
PEP	M+0	0.97	0.003	0.97	0.003	0.03	0.009	0.02	0.002
PEP	M+1	0.03	0.003	0.03	0.003	0	0	0	0
PEP	M+2	0	0	0	0	0	0	0	0
PEP	M+3	0	0	0	0	0.97	0.009	0.98	0.002
AKG	M+0	0.03	0	0.02	0.002	0.94	0.003	0.96	0.001
AKG	M+1	0	0	0	0	0.05	0.001	0.01	0
AKG	M+2	0.01	0.013	0	0	0.01	0.003	0.03	0.003
AKG	M+3	0.02	0.003	0.03	0.002	0	0	0	0.001
AKG	M+4	0.03	0.004	0.03	0.003	0	0	0	0
AKG	M+5	0.91	0.012	0.92	0.004	0	0.001	0	0.001
Glutamine	M+0	0.01	0.001	0.01	0.004	0.95	0	0.95	0
Glutamine	M+1	0	0	0	0	0.05	0	0.05	0
Glutamine	M+2	0	0	0	0	0	0	0	0
Glutamine	M+3	0	0	0	0	0	0	0	0
Glutamine	M+4	0.04	0	0.01	0.01	0	0	0	0
Glutamine	M+5	0.95	0.001	0.98	0.01	0	0	0	0
Glutamate	M+0	0.03	0.002	0.02	0.007	0.93	0.001	0.93	0.001
Glutamate	M+1	0	0	0	0	0.05	0.001	0.05	0.001
Glutamate	M+2	0	0	0	0	0.02	0	0.02	0.001
Glutamate	M+3	0.02	0	0.02	0.001	0	0	0	0
Glutamate	M+4	0.03	0.001	0.01	0.01	0	0	0	0
Glutamate	M+5	0.92	0.002	0.95	0.02	0	0	0	0
Aspartate	M+0	0.13	0	0.13	0	0.82	0.003	0.83	0.003
Aspartate	M+1	0.01	0.001	0.01	0.001	0.04	0.001	0.04	0.001
Aspartate	M+2	0.03	0.001	0.03	0.001	0.03	0	0.03	0
Aspartate	M+3	0.23	0.002	0.23	0.002	0.11	0.002	0.1	0.002
Aspartate	M+4	0.6	0.003	0.6	0.003	0	0	0	0
CarAsp	M+0	0.12	0.01	0.14	0.003	0.84	0.017	0.83	0.01
CarAsp	M+1	0.01	0.003	0.01	0.004	0.05	0.008	0.04	0.005
CarAsp	M+2	0.02	0.004	0.03	0.01	0	0	0.01	0.006
CarAsp	M+3	0.19	0.012	0.21	0.01	0.11	0.009	0.12	0.01
CarAsp	M+4	0.66	0.02	0.61	0.003	0	0	0	0
CarAsp	M+5	0	0.001	0	0	0	0	0	0
Malate	M+0	0.12	0.003	0.14	0.005	0.85	0.009	0.82	0.005
Malate	M+1	0.01	0.001	0.01	0	0.04	0.001	0.04	0.002
Malate	M+2	0.02	0.002	0.03	0.001	0.02	0.001	0.03	0.001
Malate	M+3	0.2	0.001	0.22	0.003	0.09	0.009	0.11	0.004
Malate	M+4	0.65	0.004	0.6	0.001	0	0	0	0
R5P	M+0	0.95	0.008	0.94	0.003	0	0.004	0	0.001
R5P	M+1	0.05	0.008	0.05	0.003	0	0	0	0
R5P	M+2	0	0	0.01	0	0.01	0.002	0.01	0.003
R5P	M+3	0	0	0	0	0.01	0.002	0.01	0.003
R5P	M+4	0	0	0	0	0.05	0.002	0.05	0.002
R5P	M+5	0	0	0	0	0.93	0.01	0.93	0.01
6phosphogluconate	M+0					0.06	0.038	0.02	0.004
6phosphogluconate	M+1					0	0.002	0	0
6phosphogluconate	M+2					0	0	0	0
6phosphogluconate	M+3					0	0	0	0
6phosphogluconate	M+4					0	0.001	0	0.001
6phosphogluconate	M+5					0.1	0.003	0.13	0.01
6phosphogluconate	M+6					0.84	0.04	0.85	0.01

Supplementary Table 15. Quantifying metabolite concentrations using chemical standard.

Mixture 1 (concentration of metabolites in media solution)

Compound	Cat.No. (Manufacturer)	Concentration, mM
L-Alanine	41-239-25 (Biological Industries)	50
L-Aspartic acid	41-216-25 (Biological Industries)	25
Citric acid (monohydrate)	C7129-100G (Sigma-Aldrich)	50
Fumaric acid (disodium salt)	F1506-25G (Sigma-Aldrich)	50
D-(+)-Glucose	G8720-100G (Sigma-Aldrich)	50
L-Glutamic acid	41-217-25 (Biological Industries)	50
L-Glutamine	41-218-25 (Biological Industries)	50
L-(+)-Lactic acid	L1750-10G (Sigma-Aldrich)	50
Pyruvic acid (sodium salt)	P2256-25G (Sigma-Aldrich)	50
Succinic acid (disodium salt hexahydrate)	S2378-100G	50

Mixture 2 (concentration of intercellular metabolites)

Compound	Cat.No. (Manufacturer)	Concentration, mM
Adenosine diphosphate (sodium salt)	A2754-100MG (Sigma-Aldrich)	50
L-Alanine	41-239-25 (Biological Industries)	50
L-Asparagine (monohydrate)	41-215-25 (Biological Industries)	50
L-Aspartic acid	41-216-25 (Biological Industries)	25
Adenosine triphosphate (disodium salt)	A26209-1G (Sigma-Aldrich)	50
Guanosine diphosphate (sodium salt)	G7127-10MG (Sigma-Aldrich)	50
L-Glutamic acid	41-217-25 (Biological Industries)	50
L-Glutamine	41-218-25 (Biological Industries)	50
Guanosine triphosphate (disodium salt)	G8877-10MG (Sigma-Aldrich)	50
L-Proline	41-221-25 (Biological Industries)	50

Mixture 3 (concentration of intercellular metabolites)

Compound	Cat.No. (Manufacturer)	Concentration, mM
Citric acid (monohydrate)	C7129-100G (Sigma-Aldrich)	50
Fumaric acid (disodium salt)	F1506-25G (Sigma-Aldrich)	50
α-Ketoglutaric acid (disodium salt hydrate)	K3752-5G (Sigma-Aldrich)	50
L-(−)-Malic acid	M1000-100G (Sigma-Aldrich)	50
Oxaloacetic acid	O4126-1G (Sigma-Aldrich)	50
Succinic acid (disodium salt hexahydrate)	S2378-100G (Sigma-Aldrich)	50
Succinyl-coenzyme A (sodium salt)	S1129-5MG (Sigma-Aldrich)	50

Mixture 4 (concentration of intercellular metabolites)

Compound	Cat.No. (Manufacturer)	Concentration, mM
Acetyl-coenzyme A (sodium salt)	A2056-5MG (Sigma-Aldrich)	50
D-Glucose 6-phosphate (sodium salt)	G7879-500MG	50
DL-Glyceraldehyde 3-phosphate (sodium salt)	G5251-25MG	50

Phosphoenolpyruvic acid (potassium salt)	10108294001 (Merck)	50
D-(-)-3-Phosphoglyceric acid (disodium salt)	sc-214793 (Santa Cruz)	50
6-Phosphogluconic acid (trisodium salt)	P7877-100MG	50
D-Ribose 5-phosphate (disodium salt hydrate)	R7750-10MG	50

Mixture 5 (concentration of intercellular metabolites for HILIC method)

Compound	Cat.No. (Manufacturer)	Concentration, mM
L-Alanine	41-239-25 (Biological Industries)	50
L-Asparagine (monohydrate)	41-215-25 (Biological Industries)	50
Fumaric acid (disodium salt)	F1506-25G (Sigma-Aldrich)	50
L-(−)-Malic acid	M1000-100G (Sigma-Aldrich)	50

Mixture 6 (concentration of intercellular metabolites for HILIC method (SIM)

Compound	Cat.No. (Manufacturer)	Concentration, mM
Acetyl-coenzyme A (sodium salt)	A2056-5MG (Sigma-Aldrich)	0.05
Coenzyme A (sodium salt hydrate)	C4780-25MG (Sigma-Aldrich)	0.05
Phosphoenolpyruvic acid (potassium salt)	10108294001 (Merck)	0.05
6-Phosphogluconic acid (trisodium salt)	P7877-100MG	0.25
Pyruvic acid (sodium salt)	P2256-25G (Sigma-Aldrich)	50
Succinic acid (disodium salt hexahydrate)	S2378-100G (Sigma-Aldrich)	0.25
Succinyl-coenzyme A (sodium salt)	S1129-5MG (Sigma-Aldrich)	0.05
